# Supplementary material for: Respectful maternity care delivered within health facilities in Bangladesh, Ghana and Tanzania: a cross-sectional assessment preceding a quality improvement intervention
Source: BMJ Open. 2021 Jan 20;11(1):e039616. doi: 10.1136/bmjopen-2020-039616 (PMC7818820; doi:10.1136/bmjopen-2020-039616)
Supplement: Author's manuscript [file bmjopen-2020-039616.draft_revisions.pdf]

BMJ Open is committed to open peer review. As part of this commitment we make the peer review history of every article we publish publicly available.

When an article is published we post the peer reviewers' comments and the authors' responses online. We also post the versions of the paper that were used during peer review. These are the versions that the peer review comments apply to.

The versions of the paper that follow are the versions that were submitted during the peer review process. They are not the versions of record or the final published versions. They should not be cited or distributed as the published version of this manuscript.

BMJ Open is an open access journal and the full, final, typeset and author-corrected version of record of the manuscript is available on our site with no access controls, subscription charges or pay-per-view fees (http://bmjopen.bmj.com).

If you have any questions on BMJ Open's open peer review process please email info.bmjopen@bmj.com

Respectful Maternity Care delivered within health facilities in Bangladesh, Ghana and Tanzania - A cross-sectional assessment preceding a quality improvement intervention

Journal:	*BMJ Open*
Manuscript ID	bmjopen-2020-039616
Article Type:	Original research
Date Submitted by the Author:	20-Apr-2020

Complete List of Authors:	Manu, Alexander; University of Ghana College of Health Sciences, Epidemiology and Disease Control/School of Public Health; Liverpool School of Tropical Medicine, Centre for Maternal and Newborn Health<br>Zaka, Nabila; UNICEF<br>Bianchessi, Christina; UNICEF,<br>Maswanya, Edward; NIMR<br>Williams, John; Navrongo Health Research Centre<br>Arifeen, Shams E.; ICDDR B, Maternal and Child Health Division (MCHD)

Keywords:	Quality in health care < HEALTH SERVICES ADMINISTRATION & MANAGEMENT, Epidemiology < INFECTIOUS DISEASES, Maternal medicine < OBSTETRICS

SCHOLARONE™ Manuscripts

I, the Submitting Author has the right to grant and does grant on behalf of all authors of the Work (as defined in the below author licence), an exclusive licence and/or a non-exclusive licence for contributions from authors who are: i) UK Crown employees; ii) where BMJ has agreed a CC-BY licence shall apply, and/or iii) in accordance with the terms applicable for US Federal Government officers or employees acting as part of their official duties; on a worldwide, perpetual, irrevocable, royalty-free basis to BMJ Publishing Group Ltd ("BMJ") its licensees and where the relevant Journal is co-owned by BMJ to the co-owners of the Journal, to publish the Work in this journal and any other BMJ products and to exploit all rights, as set out in our licence.

The Submitting Author accepts and understands that any supply made under these terms is made by BMJ to the Submitting Author unless you are acting as an employee on behalf of your employer or a postgraduate student of an affiliated institution which is paying any applicable article publishing charge ("APC") for Open Access articles. Where the Submitting Author wishes to make the Work available on an Open Access basis (and intends to pay the relevant APC), the terms of reuse of such Open Access shall be governed by a Creative Commons licence – details of these licences and which Creative Commons licence will apply to this Work are set out in our licence referred to above.

Other than as permitted in any relevant BMJ Author's Self Archiving Policies, I confirm this Work has not been accepted for publication elsewhere, is not being considered for publication elsewhere and does not duplicate material already published. I confirm all authors consent to publication of this Work and authorise the granting of this licence.

For peer review only

Respectful Maternity Care delivered within health facilities in Bangladesh, Ghana and Tanzania - A cross-sectional assessment preceding a quality improvement intervention

Alexander Manu[1,2], Nabila Zaka[3], Cristina Bianchessi[3], Edward Maswanya[4], John Williams[5] and Shams El Arifeen[6]

[1]Centre for Maternal and Newborn Health, Liverpool School of Tropical Medicine, Liverpool, United Kingdom
[2]School of Public Health, University of Ghana, Legon, Accra
[3]United Nations Children's Fund/HQ, New York, United States of America
[4]National Institute for Medical Research, Dar es Salaam, Tanzania
[5]Navrongo Health Research Centre, Navrongo, Ghana
[6]International Centre for Diarrhoeal Diseases Research, Bangladesh, Dhaka, Bangladesh

For peer review only

Address of authors:

NZ – nzaka@unicef.org
AM – alexander.manu@lshtm.ac.uk
CB - c.bianchessi@gmail.com
EM - edward19631@hotmail.com
JW - John.Williams@navrongo-hrc.org
SA - Shams@icddrb.org

‡Correspondence to:
Dr Alexander Manu
Liverpool School of Tropical Medicine
Pembroke Place, Liverpool.
L3 5QA. United Kingdom
Email: makmanu128@gmail.com

Keywords: Respectful care, Dignified care, Quality, Maternal. Newborn, Health facility.

Word Count: 4988

ABSTRACT

Objectives: To assess respectful maternity care (RMC) in health facilities caring for women in labour prior to implementing UNICEF's Every Mother Every Newborn Quality Improvement.

Design: Descriptive cross-sectional study involving direct observations, records review, and semi-structured interviews.

Setting: Hospital and health centres across 15 districts in Bangladesh, Ghana and Tanzania.

Participants: Forty-three facility managers, 325 providers, 849 recently-delivered women were interviewed, and 641 client-provider interactions observed.

Assessment (Intervention):Trained social-scientists and clinicians collected uniform data to assess RMC(emotional support, respectful, dignified care and communication) including policies, training, practices and experiences of women. They also checked availability of clean maternity clients' toilets and handwashing facilities.

Primary outcome: RMC provided and/or experienced by women.

Results: Between 30%-60% of providers were not trained in RMC. RMC policies and processes existed in only few facilities. In Ghana where 81%(13) of facilities had these, abuse reporting was commoner(7%), women had improved emotional support and examination findings were communicated. Maternity clients' toilets were unavailable or unclean; structural challenges prevented privacy during care and birth companion use. RMC training was rarely done as up to 80% providers could not cite two abuse prevention measures and 80%-90% did not know their facilities' client abuse response plans. Whilst up to 97% in all three countries reported satisfaction with care experienced, with Bangladesh reporting 97%(287/387), only 126(43%) will return to the same facilities for future childbirth($p<0.001$).

Conclusions: There are substantial gaps in RMC provided and consequently experienced by women in the three countries prior to EMEN-QI but provided good foundations for improvement. Providers over-rated respect and dignity in the care provided beyond what was directly observed. Asking women to rate satisfaction with care experienced may be unreliable; we found a true indicator may be the significant proportion of women who, based on their experiences, will not return to these facilities for future deliveries and must be tested.

.

For peer review only

Article Summary

Strengths and limitations of this study

- The first key strength of this study is that it used harmonised methods and developed common tools/instruments to assess respectful maternity care in the 43 health facilities across the three countries. This makes it one of the largest multi-country facility assessments from which data is amenable to direct cross-country comparisons as well as context-relevant conclusions.

- Secondly, the study employed a variety of approaches including direct observations, interviews and records review, to assess respectful care provided in the past and what is being provided at present. Whilst observations of care providers may result in the Hawthorne effect, the use of the multiple approaches and triangulation of the findings increased the objectivity. It also improved understanding of how prevailing practices measure up to the standards at individual, structural and policy levels according to Freedman et al's framework[1].

- We had limitations: the cross-sectional design and the short duration of contact with facilities allowed for assessment of limited aspects of respectful care. However, it was the most pragmatic design for many settings if data collection should not unduly interrupt caregiving.

- Also, women's reported experiences and caregiver's practices may introduce respondent biases. Observing provider-client interactions may likely find improved quality of care than what is routinely provided to clientele (Hawthorne effect). Consequently, inadequacies identified during the observations may under-represent the true extent of sub-optimal provision of respectful care.

- The findings may have limited external validity due to UNICEF's equity focus in selecting areas of work in the respective countries. Most, but not all, of the assessed facilities were in the most under-served districts and this may represent a selection bias in the context of the country.

For peer review only

INTRODUCTION

In 2015, World Health Organization (WHO) issued a statement calling for global actions to prevent and eliminate disrespect and abuse during facility-based childbirth and emphasized women's right to dignified and respectful care throughout pregnancy and childbirth[2]. Facility birth rates have increased due to increased awareness, demand and access to maternity services[3]. Approximately 6.5 million more women delivered with skilled providers in 2015 and, across 60 Countdown to 2030 countries, the median skilled attendance rate increased from 65% in 2015 to 77% in 2017[4]. However, optimism around the increasing trend in facility deliveries must be cautious because deaths among pregnant women and their babies (born and unborn) have declined at a much slower pace, confirming that, unless coupled with care of the requisite quality, outcomes for women and their babies may be poor[5-9] and communities may lose trust in facilities[10,11].

D'Oliveira et al[12] and other authors found that pregnant women were abused within health facilities during childbirth, the care they are provided is not respectful or dignified[12-14]and places no value on their expectations. The WHO framework of 2016 therefore defined eight standards for improving the quality of maternal and newborn care (MNC) in health facilities along two domains – provision and experience of care[15]. It recognises the need to amplify the voices of women from low- and middle-income settings (LMICs) in the design, content and delivery of the care they receive around childbirth to ensure that it respects their dignity, preferences and aspirations[16]. The eight WHO standards were not systematically tested in any LMIC. Moreover, it is not fully described how respect and dignity of care can be objectively measured.

As part of a Mother and Baby Friendly Hospital Initiative (MBFHI), UNICEF and partners in the Ministries of Health of Bangladesh, Ghana and Tanzania are working within selected high-mortality districts to effectively improve breastfeeding and MNC outcomes. The partnership is using this platform to implement a quasi-experimental pre-post study to test the feasibility of implementing the WHO standards within these LMICs and to answer these critical questions: how pervasive is disrespectful and abusive care within health facilities in LMICs? Do facilities have and enforce conducive policy environments and/or guidelines that enable provision and experience of respectful maternity care (RMC) and what are (and can we rely on) women's perspectives on the dignity around the care they experience? Tools such as the Mothers on Respect index have not been tested in LMICs[17].

Our objective was to measure RMC from provider perspectives and as experienced by clients to labour wards in the three countries. We also aimed to assess the implications of these on women's satisfaction with the care and interpretations of how it is measured. The manuscript therefore presents findings from a baseline assessment describing existing provisions, health worker delivery and families' perceptions of RMC prior to the implementation of the standards. The results will contribute to the continuing global discussions around how to effectively measure respect and dignity in the provision and experience of maternity care.

METHODS

Study Design and Setting

For peer review only

This is a cross-sectional study, nested within the quasi-experimental pre-post evaluation of the Every Mother Every Newborn Quality Improvement (EMEN-QI) intervention. The protocol for the evaluation of the EMEN-QI intervention has been published[18]. This study had four components: inventory to check the availability of clean toilet and handwashing facilities, policies, protocols and guidelines for providing RMC; questionnaire and qualitative interviews with facility staff and maternity clients; structured observation of maternity care provided to clients and review of client medical records for documented care.

It was carried out by researchers from three research institutions from the three countries– International Center for Diarrhoeal Diseases Research in Bangladesh (ICDDR,B), Navrongo Health Research Centre (NHRC), Ghana Health Service and the National Institute for Medical Research (NIMR), Tanzania. It was conducted between May and August 2016 in 43 health facilities in Bangladesh (15), Ghana (16) and Tanzania (12), comprising 1 regional and 12 district hospitals and 30 health centres (HCs)/upazilla health complexes (UHCs). Teams of 2-4 clinicians (CRAs) and research social scientists/anthropologists (RSAs) carried out the assessment in each facility. These teams were trained by the principal investigators in each country under the direct supervision of the lead author. The training was followed by pretesting exercises which included hospital visits until there was consensus on the observations made for client-provider interactions.

In Bangladesh, three hospitals and twelve UHCs in Kurigram, Lalmonirhat and Gaibandha districts of the Rangpur division were involved. In Ghana, the regional hospital of the Upper East Region (UER), five district hospitals and ten health centres in eight districts (Bawku, Kassena-Nankana East and Bolgatanga Municipalities and Bawku West, Bongo, Kassena-Nankana West and Builsa North Districts) were covered. For Tanzania, the assessments were done in four hospitals and eight health centres within Njombe Town Council, Makete, Ludewa and Wanging'ombe districts of the Njombe region. These facilities provide basic/comprehensive emergency obstetric care (BEmOC/CEmOC) services and were selected in consultation with the ministries of health of the respective countries to reflect high average caseload, relatively poor maternal and newborn health indices and nationally-representative human resource availability.[18] All district hospitals, UHCs in Bangladesh and one HC Tanzania provided CEmOC services.

Participants

To cover provision and experience of care, respondents for the study included facility managers and "in-charges", providers who directly cared for women in labour for the two weeks of the assessment, recently-delivered women and their companions. The health records of all women who were seen and cared for at the maternity in the 3-6 months prior to the assessment were reviewed and data abstracted.

Informed Consent

We consented all participants to participate in the study. Research Assistants read out or gave an information sheet to all participants explaining the purpose of the assessment in a language of their choice. Each participant asked for clarifications on the study. They were informed that there will be no direct benefits for participation but the information they provide will help improve the quality of maternity care provided in facilities. They were assured of their right to withdraw participation at any time without affecting their position in the facility or care they received and that information provided

For peer review only

will be treated as confidential. Agreement to participate was indicated with a signature/thumbprint. No respondent refused participation in the study. All interviews lasted 45-60 minutes.

Ethics Approval

The study protocol was approved by institutional ethics review boards (IRBs) in all three countries: ICDDR,B in Bangladesh (PR-16024, 1st June 2016), Ghana Health Service (GHS) in Ghana (NHRCIRB226, 6th April 2016) and NIMR in Tanzania (NIMR/HQ/R.8a/Vol. IX/2176, 11th April 2016). Clearance was sought from the facility managers for the study in the respective facilities.

Patient and Public Involvement

The study tools and instruments were pretested in all the three countries to solicit facility-user input into the strategy for data collection. The data collection involved patients or maternity clients who have been discharged from the facilities as well as their companions who accompanied them to the facility for the delivery. Their identities were protected, and their confidentiality and privacy were ensured all the information they provided. Their contributions have been cited in the acknowledgements.

Assessment of Respectful Maternity Care

Measurement of Respectful maternity care (RMC): RMC was measured along the dimensions of the three components in the WHO framework - emotional support, respectful communication and respectful and dignified care - from both the perspectives of the provider, the women (clients) and an independent informed assessor (research clinicians or CRAs). These were supplemented with data on the availability of the right policy environment and facility provisions (e.g. handwashing) for RMC.

After facility entry, CRAs interviewed 43 facility superintendents/"in-charges" and assessed whether the facility had specific protocols and guidelines for ensuring RMC including staff training at maternity units. Using a semi-structured questionnaire, the CRAs assessed whether facilities had systems for identifying abuses of pregnant women seeking or receiving care including "whistle blower" policies where care providers could report client abuses perpetrated by their peers under protected or concealed identities. Respondents were asked how they involved communities in defining and providing quality and woman-centred maternity care. We considered the availability of clean client toilets on the maternity as a basic necessity in providing respectful care and so CRAs checked whether maternity wards had toilets and rated their cleanliness based on agreed criteria.

CRAs also 325 interviewed care providers in the maternity wards on any formal RMC training they received, any encounter with situations of client abuse in facilities and management responses to abuses. Care providers included specialists, doctors, midwives and staff nurses. Selection was biased towards staff who play key leadership or decision-making roles in facilities (e.g. matrons who provide care and enforced guidelines and regulations) or served for more than six months.

The CRAs also passively 641 observed client-provider interactions as women navigated through all the points of care provision within facilities–from reception through till discharge after delivery. This provided first-hand experience of auditory and visual privacy and provider-client communication including explanation of the care plan and the involvement of clients in their own care. They checked whether providers ensured a conducive atmosphere for women and their companions to ask

questions on the care they received; support (emotional and other) for women during childbirth; and any abuses. They reviewed women's records to assess documentation of care.

RSAs identified isolated, comfortable and conducive spaces within facility to interview 829 recently-delivered women who have been discharged from facilities on their experience of maternity care provided. All women who have been in the facility for at least six hours and consent to participate were interviewed irrespective of birth outcomes. This was because women who present in labour are observed for at least 6 hours after the birth before discharge, creating opportunity for them to interact with providers. The RSAs ensured that respondents were not seen or heard during the interview. The interview covered women's perspectives on provider communications, responsiveness, respect for their choices and preferences, visual and auditory privacy during care provision, demand for informal or formal payments and withholding of care if such demands were refused, any physical, sexual or verbal abuses during women's stay in the facility. Clients' impressions and satisfaction with the maternity care they experienced were also elicited but aside that, they were asked whether their experiences will make them recommend the facility to relatives or friends for maternity care or whether they themselves will like to return to the same facility for their next childbirth.

RSAs also interviewed any persons accompanying the women during their stay in the facility for their independent perspectives on the care provided to their relative or friend. RSAs asked whether providers demanded or collected payments for services or whether any service was withheld for any reason including inability to pay.

Quality assurance and data processing

Stringent uniform quality assurance (QA) processes were implemented starting from the field through to the data processing units of the research institutions. Team coordinators liaised between fieldworkers, facility leadership and data centres. Data collection was paper-based in Ghana and Bangladesh but electronic in Tanzania. At the end of each day, research teams met and peer-reviewed each other's forms for completeness. Team coordinators re-checked all forms to ensure blanks or inconsistencies were identified and resolved. All checked forms were batched, logged into batch control sheets and transmitted to computer centres for processing.

In Ghana and Bangladesh, two data entry clerks independently entered the data and the two data streams were compared for agreement. Any disagreements were resolved with the source questionnaire by data managers who then run range and consistency and data integrity checks to generate exception reports for the study investigators to review and resolve. In Tanzania, data were directly transmitted to central servers for review by data managers. Where required, forms with challenges were sent back to the field for resolution. Cleaned data were securely stored on password-protected servers. A copy of the final data was shared with UNICEF/HQ.

Data analyses

Data were transferred to Stata version 14.1 for analyses. Data were represented with tabular, numerical and graphical methods. Proportions, means(sd) and medians (IQR) were estimated for the outcome analyses. Responses to open-ended questions were coded into themes in NVIVO. These were triangulated to the close-ended questions to facilitate their contextual interpretations.

Coordination

For peer review only

UNICEF coordinated the uniform assessment across countries. A consultant (AM) facilitated a UNICEF-organised design meeting and training in New York with principal investigators (PIs). Skype conference calls were organized every week to agree harmonised timelines for implementation and update on progress. The coordinated approach allowed for common solutions to challenges encountered and for the consultant to visit each country during the training and initiation of data collection.

RESULTS

Health facility managers in all 43 facilities across the three countries were interviewed. There were 325 care provider interviews with nurses, midwives and doctors comprising 158 in Bangladesh, 86 in Ghana and 81 in Tanzania. Questionnaires were administered by social scientists/anthropologists to collect data from a total of 849 recently-delivered women: 295 from Bangladesh, 381 from Ghana and 173 from Tanzania. Observation of 641 client-provider interactions was conducted by CRAs – 387 in Bangladesh, 134 in Ghana and 120 in Tanzania.

Availability of dedicated clean maternity toilet as part of respectful maternity care

Clean toilets with handwashing facilities were inadequate in the maternity units in all three countries (figure 1). Between 20%-25% of facilities did not have separate toilet facilities for women on the maternity wards. Where they existed, they were mostly (from 6/12=50% in Tanzania to 15/15=100% in Bangladesh) judged to be unclean and from 8/16=50% in Ghana to 12/15=80% in Bangladesh were without soap and water for handwashing. Women's perceptions on the cleanliness of the toilets contrasted with that from the observations. Figure 2 shows that 104 (35%) of the 295 women interviewed in Bangladesh thought the toilets were unclean, as did 301 (79%) of the 381 women interviewed in Ghana.

Availability of the right policy environment for respectful maternity care

Between the three countries, health facilities in Ghana were the most likely to have established processes for identifying and reporting client abuses (Table 1); facilities in Tanzania being the least likely. Majority (13/16=81.3%) of facility managers in Ghana, compared to 11 (73.3%) in Bangladesh and 6 (50.0%) in Tanzania, reported the existence of clear processes for identifying client abuses. In Ghana, all but one facility had a "whistle-blower" policy for reporting abuses, and all facilities (100%) actively encourage women to report any provider abuses. In Tanzania only 1 in 4 facilities had a "whistle-blower" policy and one in three encourage women to report abuses. Data on whistle blower policies were not captured in Bangladesh although some staff suggested that mechanisms for making anonymous complaints using mobile SMS are captured into the district health information systems (DHIS-2) platform and followed-up regularly.

Consistent with facility managers' reports, assessors found that health facilities in Ghana were the most likely, of all three countries, to have policies on women's rights during care at the facility. Many (61/86=71%) Ghanaian facilities also had policies on addressing patient concerns and 70 (82%) had policies on the rights of clients who sought maternity and newborn care (table 2). Facilities in Bangladesh reported the lowest availability of these policies (54 (41%) had policies to address client concerns and 83(53%) on the rights of client). In Bangladeshi facilities that reported that these policies existed, staff could not show copies to the assessors during the visits. In Tanzania, 54 (67%) facilities

For peer review only

had policies to address patient concerns whilst 57 (70%) had policies on their rights whilst receiving care (table 2)

Training of providers in respectful maternity care

Staff in facilities were not provided specific training on how to provide maternity care with dignity and respect to women and many were oblivious of their own facility's procedures for addressing abuses. Between 30%-60% of providers said they have not had any training on how to treat childbearing women with dignity and respect. Up to 80% of them could not mention two measures being implemented in their facilities to prevent abuses of women and babies during care. A substantial proportion (69 (80%) in Ghana and 142 (90%) in Bangladesh) did not know of two or more measures in place to treat or rehabilitate victims of abuse (table 2). Meanwhile, table 2 also shows that almost all of these providers considered that the care they provide to women around childbirth was respectful and dignified by rating the respect and dignity in the care above 3 on a scale of 1 to 5, 5 being the poorest: 151 (96%) in Bangladesh to 86 (100%) in Ghana.

Women's reported experience of maternity care and how it compares with assessors' observations

Women's account of their experiences during maternity care appeared to be inconsistent. Whilst most of them said they were happy with the attitude of providers (table 3), a good number said the provider was not doing enough for them and/or their babies and were not given the opportunity to express these concerns (figure 2). For example, in Tanzania, although 168 (97.1%) of women thought they were treated with "respect" by the providers (table 3) and 161 (93.1%) said providers were responsive to them when they asked for support, figure 2 also shows that about a quarter of them 42 (24.3%) had moments when they were worried that the providers were not doing enough for them or their babies. Moreover, over 106 (61.3%) said the providers did not even give them the opportunity to express these concerns or ask questions. The trend was not different in Ghana and Bangladesh. In Bangladesh, for instance, where 266 (90.2%) of women said they were treated with respect, about 106 (35.9%) had moments they were concerned that the providers were not doing enough for them and 87 (29.5%) thought they were not given the opportunity to express these concerns.

The reasons for the apparent inconsistency in women's report was evident during the observation of client-provider interactions by the study assessors. Their observations revealed contrasting trends between the African (Ghana and Tanzania) and Asian (Bangladesh) settings. Figures 3a and 3b show that in Ghana and Tanzania, at reception, the providers were mostly welcoming: 113 (84%) and 103 (86%) respectively; and were supportive and caring when women were in labour pain 107 (80%) and 95 (79%) respectively. The assessors also found that the providers were mostly "always courteous" in communication with the women (119/134=89% in Ghana and 108/120=90% in Tanzania), and in the two countries inform 62%-72% of women about their initial examination findings; discuss delivery plans with 52%-72% of them; reassure and allay the fears of women in over 70% of cases and confirm delivery plans during labour monitoring in 63%-71% of cases. However, figure 3c shows that, in these two countries, 12%-27% of respondents were not "comfortable" talking to the providers and 2.3%-2.5% were visibly uncomfortable or timid. We found only 3% of women in the two countries made specific requests from the providers and none in Ghana could request for a birth companion (Table 4).

For peer review only

In contrast, the statistics were lower in Bangladesh. Figures 3a and 3b show that providers were always welcoming to 149 (35.8%) of women, were supportive and caring during labour pains in 188 (48.5%) and just about half 198 (51.2%) were told examination findings at initial exams. Though the delivery plan was discussed with 282 (72.9%) of the women, during labour monitoring, these plans were confirmed with only 3(0.7%) of them. Table 4 shows that about 14 (3.7%) of women in Bangladesh made specific request from the providers and though all were refused, it was only in a single case that the refusal was done respectfully. Figure 3c shows that 25 (7%) of women in Bangladesh (close to three-times the percentage in the African settings) felt uncomfortable and timid when talking to the providers.

Companion at birth

No woman in Ghana and only two (1.7%) in Tanzania reported requesting a birth companion for the delivery (Table 4). This contrasts with the 286 (73.9%) in Bangladesh. However, in figure 3a, women's companions were allowed to be present for 17 (12.7%) of women in Ghana, 5 (4.2%) in Tanzania and 374 (96.6%) in Bangladesh.

Verbal, physical or sexual abuse of women during maternity care

Facility managers (Table 1) also shows that cases of abuse of women during maternity care were not uncommon but measures to prevent recurrence were weak. Facility managers were aware of recent (less than 2 weeks) cases of abuse in their facilities. Possibly because of advanced procedures for encouraging people to report abuses, five cases of abuse were reported to facility managers in Ghana compared to only one each in Bangladesh and Tanzania.

Women reported that they were physically, verbally or sexually abused by providers during care-giving (Table 3). The commonest form of abuse was verbal; on average, 6% of women in all three countries experienced this form of abuse (4.6% in Tanzania to 6.6% in Ghana). Sexual abuses were the rarest with only 1 or 2 women reporting the experience in each country. On average, 2% of the women also experienced physical abuses from providers during their stay in the facility.

When these abuses occurred, facilities in Ghana and Tanzania conducted internal investigations and the only action taken to prevent future occurrences was counselling or verbal warnings to the staff involved (5 of the 6 cases). In comparison, the only recent case in Bangladesh was referred to an external body for investigation and action. Although a substantial proportion of providers – 16 (20%) in Tanzania to 27 (32%) in Ghana - had encountered victims of abuse in their practice, up to 70% did not know of any existing systematic processes for identifying and reporting abuses within their facilities.

For peer review only

Privacy of care

During assessors' observations of client-provider interactions, of all the three countries, women's privacy was mostly ensured during maternity care in Tanzania (figure 3d). In Tanzania, women's privacy was ensured during 82.5% (99) of all interactions with women. During delivery care, where they scored the lowest, privacy was ensured in eight out of every ten instances during CRA observations. This was in sharp contrast to Ghana where privacy was ensured in only 28 (20.9%) of all interactions with women. Women's privacy was mostly considered in Ghana during labour monitoring on the wards (92 (69%) of instances). Bangladeshi facilities were between the two extremes with privacy ensured at all times for 139 (36.0%) and during labour and delivery for 218 (56.3%) of women.

Demand for payment and withholding of delivery care services for inability to pay

Consistent with findings from CRA observations of client-provider interactions, providers in all three countries also reported that women are made to pay monies for supplies and/or delivery, even in obstetric emergencies (table 2). Charging fees was most common within facilities in Tanzania where 42 (52%) providers said women pay fees for normal deliveries. A third (27) of the providers in Tanzania also said these payments were also expected for obstetric emergencies. The proportion of providers reporting these payments were relatively lower in Bangladesh where 32 (20%) reported payments for normal deliveries and 19 (12%) in emergencies. In Ghana, where a health insurance scheme covers the cost of facility births, 7 (8%) providers reported that fees were still charged for normal deliveries and 9 (10%) for obstetric emergencies. In all three countries, the majority of the facility providers reported that women were expected to pay for delivery supplies. From a high of 73 (85%) of providers in Ghana, 132 (83%) in Bangladesh to 61 (75%) in Tanzania, providers reported that women had to buy delivery supplies in the facility.

Study assessors also found providers demanding monies from women for maternity services and supplies. this practice was most common in Tanzania where women paid for services in 61 (51%) cases observed. In Bangladesh, the proportion was 164 (42%) but only 19 (14%) in Ghana. Maternity services were sometimes withheld from women when they are unable to pay and, whilst some complained about the payments in general, they were in the minority. During observations, only a single mother in Ghana, 45 (12%) in Bangladesh and 22 (18%) in Tanzania were seen complaining about the charges. It is noteworthy that some delivery care services were withheld from women (11% in Tanzania and 0.5% in Bangladesh) because they were not able to pay these monies. No service was withheld in Ghana on account of women's inability to pay.

Community involvement providing respectful maternity care

Facilities do not often involve community stakeholders in quality of care considerations, according to managers' reports. One-half of all facilities in Tanzania (6; 50%) and Bangladesh (8; 53%) involved community stakeholders in deliberations around ensuring quality maternity and newborn care, lower than the 75% (12) in Ghanaian facilities (table 2). When care is abusive or not respectful, less than a third of facilities in Bangladesh (49; 31%) and Tanzania (27; 33%) involve community stakeholders in investigations/ corrective strategy formulation (table 3). In Ghana, more than half (49/86) of the facilities did so.

Overall satisfaction with the maternity care at health facilities

For peer review only

Care providers thought that the care they provided to women was with dignity and respect, but mothers and families seemed to have different perceptions on the care they experienced. All the 325 care providers who participated in the interviews were asked to rank their facilities on a scale of 1 to 5 (5 being the best) in terms of RMC they provide to women. All 86 providers from Ghana, 151 (95.6%) from Bangladesh and 77 (95.1%) from Tanzania ranked their facilities 3 and above. Based on women's experiences, over 90% of them said they were satisfied with the attitude of the providers - 271 (91.7%) in Bangladesh, 353 (92.7%) in Ghana and 161 (93.1%) in Tanzania (Table 3). A lower proportion of women/caregivers were satisfied with the information they were provided after the birth with regards to breastfeeding (64.8%-88.7%) and family planning (12.9%-54.6%), and the differences in these proportions (provider attitude and information giving) were statistically significant: breastfeeding; differences=4.0%-36.9%; $p<0.03$ and family planning; differences=38.1%-78.8%, $p<0.001$.

Figure 4, however, shows that although majority of women expressed satisfaction with the respect and dignity in the maternity care they received at facilities and will recommend these facilities to their relatives or friends, based on their experiences, they themselves would not return to the same facility for maternity care in their next delivery. Women in Bangladesh were the most likely to express satisfaction with care received (287; 97%) but, on the contrary, were the least likely 106 (43%) to return to the same facilities for care: $p<0.001$ for the difference in proportions. Similarly, only 305 (80%) in Ghana and 126 (73%) in Tanzania will return to the same facilities in the future for maternity care and these differences were statistically significant: $p<0.0001$. In Tanzania compared to Bangladesh and Ghana, many more women would be willing to recommend a facility to a relative or friend for maternity care even if they themselves were not satisfied with the care they received; 152 (88%) satisfied with care and 157 (91%) will recommend facility to a relative or friend – although the difference was not statistically significant ($p=0.21$).

DISCUSSION

Respectful maternity care is a relatively new and developing concept in many LMICs. The experience of the three components: respectful and dignified care, emotional support and respectful communication by women who went for maternity care at assessed health facilities in Bangladesh, Ghana and Tanzania prior to the onset of the UNICEF/WHO quality improvement interventions had many good components but also substantial gaps. Creation of the right policy environment for providing RMC, like in Ghana, was associated with more substantial advancements in the provision and women's experience of RMC including the ease of women to report abuses when experienced in the facility. Supportive care starts when providers warmly welcome women at reception, and in these instances, women rated their overall experiences better. Communication was poor as examinations were done but women were not told of the findings or the implications for their care. Many women were not allowed to express opinions in the care they received but even where they did, they felt their concerns were not taken into consideration during their care. There are some components of RMC such as privacy and use of birth companions that were difficult to implement because of the set-up in the maternity units of these facilities. The use of a variety of methods for the measurement of RMC provided internal validation of the findings that would not have been achievable if any of the methods were implemented in isolation.

Measuring satisfaction with care with questions that merely ask whether women or providers were satisfied with care received or provided was confirmed to be unreliable[10]. Our results show that whilst

For peer review only

88%-97% of women and providers highly rated their satisfaction with care received or provided, perhaps a better index of satisfaction with care is the significant 20% to 58% ($p<0.0001$) of women who reported that, based on the maternity care experienced, they will not return to the same facilities for birth in the future. This apparent inconsistency between women's reported satisfaction with care and desire to return to same facility has not often been measured although alluded to when researchers find unbelievably high reported level of satisfaction under situations that did not appear to merit same[10,11]. Lack of respectful and dignified care are known barriers to subsequent careseeking[19].

There appeared differences in observed provider attitudes between the South Asian (SA) and sub-Saharan African (SSA) facilities in that, apart from Bangladesh, Figure 3a shows that providers were mostly welcoming to women and supported them during labour pains. With only 38.5% of providers welcoming women and 48.3% supporting in Bangladesh compared to over 80% Ghana or Tanzania, it was not surprising that about 7% of women were intimidated to talk to providers. Meanwhile, 17%-36% of women across the three countries were sometimes concerned that the providers were not providing enough care for them but could not express. The UK quality care commission[20] defines respect and dignity in care as care "that treats care recipients as equals" who merely require support from care providers to be autonomous, independent[21]. This definition conforms with 4th, 5th and 6th of the WHO standards for facility quality of care for mothers and newborns[15].

Abuse is a seen as a way of controlling clients to undermine their autonomy[12]. The assessment findings confirm that, across the three countries, physical, sexual, verbal and psychological abuses (where services are withheld from women because of inability to pay informal/illegal charges) were not uncommon. These findings concur with reports of care providers "scolding" or "shouting at" women and verbalising words that were "unkind, brusque, rude, unsympathetic and uncaring" to them[22,23]. We believe that the 4.6%-6.6% of verbal abuses may represent gross under-reporting and that challenge might be bigger. Also, providing RMC requires development of infrastructure. For instance, none of the three countries prohibits the use of birth companions but the set-up of the units do not allow for privacy.

Respectful care is also not merely the absence of abuses. Policies on the rights of maternity clients and for addressing abuse are not always present in facilities particularly in Tanzania. Even when they exist, providers were oblivious, or the enforcement was weak. A conducive policy atmosphere facilitates reporting of abuse cases. Our finding that 25% (Tanzania) to 60% (Bangladesh) of providers have not been trained on provision of dignified and RMC is unfortunate. For instance, Ghana reported the most advanced policy environment to identify and address abuses including a "whistle-blower" policy. Consequently, women in Ghana were the most likely to report of abuses. This confirms that addressing abuses in maternity and newborn care requires deliberate efforts to create the environment that will address systems rather than individual failures that occasion "blame" – literally "A systems approach in a person-centred system"[24,25].

The WHO statement affirms every woman's right to dignified, respectful healthcare, identifying an increasing need for more research to measure the burden and pervasiveness of disrespect and abuse in health facilities[2,22,23,26]. Vogel et al[26] added that poor measurement of respectful care may result from the lack of globally applicable terminologies. Initiatives to improve respect around childbirth care are not new in these countries albeit on smaller scales: Bangladesh has implemented a Women

For peer review only

Friendly Health Services Initiative in some facilities but Kurigram district did not benefit. Similarly, the Ifakara Research Institute in Tanzania is also implementing RMC interventions in some facilities. These have not permeated the healthcare systems. That quality affects outcomes is incontrovertible[27]. We believe that efforts to provide RMC must be intentional, goal-directed, measurable, backed by a strong political will, and should have robust systems of accountability[28].

This study has many strengths: first, using harmonised methods to assess the 43 facilities makes it one of the largest multi-country facility assessments from which data is amenable to direct cross-country comparisons. Secondly, the use of a variety of approaches including observations and data review increased the objectivity and improved understanding of how prevailing practices measure up to the standards at individual, structural and policy levels according to Freedman et al's framework[1]. We had limitations: the cross-sectional design and the short duration of contact with facilities allowed for assessment of limited aspects of respectful care. However, it was the most pragmatic design for many settings if it should not unduly interrupt caregiving. Women's reported experiences and caregiver's practices may introduce respondent biases. Observing provider-client interactions may likely to make them go beyond what they usually do. Consequently, inadequacies identified during the observations may represent a small dip in an abyss of inadequate respect in care. These findings may not be generalisable to all facilities in the respective countries because, due to UNICEF's equity approach, assessed facilities were located in most under-served and least-developed districts which may imply selection biases.

This study contributes to amplifying women's voices, which is key to the values of the WHO/UNICEF-coordinated Quality, Equity, Dignity network and the recent petition on "What women want?"[29]. However, women's aversion to non-dignified and disrespectful care, nuanced in their decision not to patronise these facilities in the future should alert health systems that community trust in facilities for delivery care should not be taken for granted. The Lancet Quality Commission advocates for measurement of client satisfaction on a systematic basis[10].

In conclusion, substantial gaps exist in RMC provided and consequently experienced by women across the three countries, but there were many good practices that could form a foundation for quality improvement interventions. Health providers' accounts over-rated the respect and dignity in the care they provide to women way beyond what was directly observed.

It calls for more rigorous measurement of efficacy of these measures as well as in-depth anthropological investigation to understand the drivers of respectful and dignified care to help develop corrective strategies and accountability mechanisms around these in a participatory. As a first step, respectful care must form a critical component of pre-service training in all countries. Community engagement through organizing women groups for a systematic demand creation around respectful care, follow-up and inclusion in a holistic system response will be key. Recognising men (other community members) and involving them as partners and stakeholders will achieve much more in terms of a demand-driven or demand-guided RMC and will require some investment. Periodic client-exit interviews, use of SMS, comment boxes or easy-touch buttons for client feedback will be useful but need to be coupled with capacity to retrieve these data, systematically analyse them and use them to inform changes. Health systems and facilities should be bold to empower an approachable community ombudsman to listen to client complaints and provide systematic feedback

For peer review only

into the delivery respectful maternity care in facilities[30] . Until then, our mothers will not return to our facilities after the experiences they receive.

Author contributions

The study was designed by AM, NZ, EM, JW, and SA with technical input from CX. AM, NZ, EM, JW, SA, formed a team that managed and supported the respective countries to implement the evaluation. AM and NZ drafted the manuscript and obtained inputs from the co-authors: JW, EM, SA, CX. The revised manuscripts were proof-read by all authors who helped in finalising it. All co-authors (NZ, AM, EM, JW, SA) read the final manuscript and agreed to its submission for publication in this journal.

Acknowledgements

This work was conducted under the UNICEF-BMGF MNCH partnership. We also appreciate the contributions from Dr Priscilla Wobil (UNICEF Ghana), Masum Billah (ICDDR,B Bangladesh), Stella Kilima (NIMR, Tanzania), Francis Yeji (NHRC/GHS, Ghana), management and staff of our collaborating institutions at the International Centre for Diarrhoeal Disease Research, Bangladesh, Navrongo Research Centre of the Ghana Health Service, National Institute of Medical Research, Tanzania and the UNICEF country offices in the three countries. The multiple consultations with a wide range of stakeholders including global/international experts and professionals in maternal and newborn health, ministries of health and partners in Bangladesh, Ghana and Tanzania. We also thank the mothers who participated in the design and provided data for this analysis.

Competing interests

All the authors declare no conflict of or competing interests.

Funding

The study was funded by the Bill and Melinda Gates Foundation through UNICEF headquarters, New York. The findings and conclusions in this report are those of the authors and do not represent the official position of these organizations.

Data Sharing Statement

The data analysed for this manuscript is part of the bigger EMEN assessment being undertaken by UNICEF. Access to the data will follow site-specific and national guidelines of the three countries and agreements with UNICEF HQ.

For peer review only

References

1. Freedman LP, Ramsey K, Abuya T, et al. Defining disrespect and abuse of women in childbirth: a research, policy and rights agenda. *Bull World Health Organ* 2014; **92**(12): 915-7.
2. World Health Organization. The prevention and elimination of disrespect and abuse during facility-based childbirth. In: Department of Reproductive Health and Research WHO, editor. Geneva, Switzerland: World Health Organization; 2015.
3. Ng M, Misra A, Diwan V, Agnani M, Levin-Rector A, De Costa A. An assessment of the impact of the JSY cash transfer program on maternal mortality reduction in Madhya Pradesh, India. *Glob Health Action* 2014; **7**: 24939.
4. Countdown to 2030 Collaboration. Countdown to 2030: tracking progress towards universal coverage for reproductive, maternal, newborn, and child health. *Lancet* 2018; **391**(10129): 1538-48.
5. Kruk ME, Gage AD, Joseph NT, Danaei G, Garcia-Saiso S, Salomon JA. Mortality due to low-quality health systems in the universal health coverage era: a systematic analysis of amenable deaths in 137 countries. *Lancet* 2018; **392**(10160): 2203-12.
6. Lawn JE, Blencowe H, Waiswa P, et al. Stillbirths: rates, risk factors, and acceleration towards 2030. *Lancet* 2016; **387**(10018): 587-603.
7. Ronsmans C, Graham WJ, Lancet Maternal Survival Series steering g. Maternal mortality: who, when, where, and why. *Lancet* 2006; **368**(9542): 1189-200.
8. Doyle C, Lennox L, Bell D. A systematic review of evidence on the links between patient experience and clinical safety and effectiveness. *BMJ Open* 2013; **3**(1).
9. Souza JP, Gulmezoglu AM, Vogel J, et al. Moving beyond essential interventions for reduction of maternal mortality (the WHO Multicountry Survey on Maternal and Newborn Health): a cross-sectional study. *Lancet* 2013; **381**(9879): 1747-55.
10. Kruk ME, Gage AD, Arsenault C, et al. High-quality health systems in the Sustainable Development Goals era: time for a revolution. *Lancet Glob Health* 2018; **6**(11): e1196-e252.
11. Larson E, Leslie HH, Kruk ME. The determinants and outcomes of good provider communication: a cross-sectional study in seven African countries. *BMJ Open* 2017; **7**(6): e014888.
12. d'Oliveira AF, Diniz SG, Schraiber LB. Violence against women in health-care institutions: an emerging problem. *Lancet* 2002; **359**(9318): 1681-5.
13. Silal SP, Penn-Kekana L, Harris B, Birch S, McIntyre D. Exploring inequalities in access to and use of maternal health services in South Africa. *BMC Health Serv Res* 2012; **12**: 120.
14. Small R, Yelland J, Lumley J, Brown S, Liamputtong P. Immigrant women's views about care during labor and birth: an Australian study of Vietnamese, Turkish, and Filipino women. *Birth* 2002; **29**(4): 266-77.
15. World Health Organization. Standards for improving quality of maternal and newborn care in health facilities. Geneva, Switzwerland: World Health Organization; 2016.
16. de Bernis L, Kinney MV, Stones W, et al. Stillbirths: ending preventable deaths by 2030. *Lancet* 2016; **387**(10019): 703-16.
17. Vedam S, Stoll K, Rubashkin N, et al. The Mothers on Respect (MOR) index: measuring quality, safety, and human rights in childbirth. *SSM Popul Health* 2017; **3**: 201-10.
18. Manu A, Arifeen S, Williams J, et al. Assessment of facility readiness for implementing the WHO/UNICEF standards for improving quality of maternal and newborn care in health facilities - experiences from UNICEF's implementation in three countries of South Asia and sub-Saharan Africa. *BMC Health Serv Res* 2018; **18**(1): 531.
19. Bohren MA, Hunter EC, Munthe-Kaas HM, Souza JP, Vogel JP, Gulmezoglu AM. Facilitators and barriers to facility-based delivery in low- and middle-income countries: a qualitative evidence synthesis. *Reprod Health* 2014; **11**(1): 71.
20. Care Quality Commission UK. Health and Social Care Act 2008 (Regulated Activities) Regulations 2014: Regulation 10. http://www.cqc.org.uk/guidance-providers/regulations-enforcement/regulation-10-dignity-respect.

For peer review only

21. World Health Organization; Organisation for Economic Co-operation and Development; and The World Bank. Delivering quality health services: a global imperative for universal health coverage. Geneva, Switzerland: World Health Organization Organisation for Economic Co-operation and Development and The World Bank., 2018.
22. Bohren MA, Vogel JP, Tuncalp O, et al. "By slapping their laps, the patient will know that you truly care for her": A qualitative study on social norms and acceptability of the mistreatment of women during childbirth in Abuja, Nigeria. *SSM Popul Health* 2016; **2**: 640-55.
23. Bohren MA, Vogel JP, Hunter EC, et al. The Mistreatment of Women during Childbirth in Health Facilities Globally: A Mixed-Methods Systematic Review. *PLoS Med* 2015; **12**(6): e1001847; discussion e.
24. National Academies of Sciences Engineering and Medicine. Crossing

the global quality chasm: Improving health care worldwide. Washington, DC, USA: National Academies of Sciences, Engineering, and Medicine, 2018.
25. Buckley R. World Development Report 2004: Making services work for poor people-Overview. Washington, DC, USA: The World Bank, 2003. http://documents.worldbank.org/curated/en/527371468166770790/, 2004.
26. Vogel JP, Bohren MA, Tuncalp, Oladapo OT, Gulmezoglu AM. Promoting respect and preventing mistreatment during childbirth. *BJOG* 2016; **123**(5): 671-4.
27. Miller S, Lalonde A. The global epidemic of abuse and disrespect during childbirth: History, evidence, interventions, and FIGO's mother-baby friendly birthing facilities initiative. *Int J Gynaecol Obstet* 2015; **131 Suppl 1**: S49-52.
28. Van Belle S, Mayhew SH. Public accountability needs to be enforced -a case study of the governance arrangements and accountability practices in a rural health district in Ghana. *BMC Health Serv Res* 2016; **16**(1): 568.
29. White Ribbon Alliance. What Women Want: Demands for quality healthcare for women and girls. https://www.whiteribbonalliance.org/whatwomenwant/. Accessed November 2018.
30. World Health Organization and the United Nations Children’s Fund (UNICEF). Declaration of Astana: From Alma-Ata towards universal health coverage and the Sustainable Development Goals. Astana, Kazakhstan: World Health Organization; 2018.

For peer review only

List of tables

Table 1. Facility managers' report on the availability of guidelines for identifying, responding and preventing abuses and community involvement in ensuring quality of care

Guideline or policy parameter in health facilities	Number of responses (%)		
	Bangladesh N=15	Ghana N=16	Tanzania N=12
A process for identifying abuses of client by provider	11 (73.3%)	13 (81.3%)	6 (50.0%)
"Whistle-blower" policy for peer reporting under cover	Not recoded	15 (93.8%)	3 (25.0%)
Women are encouraged to report provider abuses	9 (60.0%)	16 (100.0%)	4 (33.3%)
Response to most recent abuse case (number of cases)	1 case	5 cases	1 case
- *Investigated and penal actions taken internally*	0 (0.0%)	4 (80.0%)	1 (100.0%)
- *Investigated within facility but referred for action*	0 (0.0%)	1 (20.0%)	0 (0.0%)
- *Referred to external bodies for investigation & sanction*	1 (100.0%)	0 (0.0%)	0 (0.0%)
Measures taken to prevent future abuses[¥]			
- *Counselling and verbal warning*	1 (100.0%)*	5 (100.0%)	1 (100.0%)
Involves community stakeholders in quality improvement efforts	8 (53.3%)	12 (75.0%)	6 (50.0%)
Women's support groups exist in the catchment population	5 (33.3%)	10 (62.5%)	2 (16.7%)

**In Bangladesh, they raise awareness and supervise the staff who perpetrated the act.* [¥]*The assessment checked for other penal measures such as demotion of staff, reassignment/transfer, embargo on salary and interdiction but there was not reported in any of the facilities.*

For peer review only

Table 2. Care providers' report on availability of guidelines for respectful maternal and newborn care

Subject area	Providers' account on modalities for ensuring respectful maternity and newborn care	Number of responses (%)		
		Bangladesh N=158	Ghana N=86	Tanzania N=81
Policies on women's rights	Policy and procedure for addressing patient concerns	54 (41.1%)	61 (71.0%)	54 (66.7%)
	Policies on the rights of patients	83 (52.5%)	70 (81.6%)	57 (70.4%)
Staff training rating of respectful maternity care	Staff trained on how to treat childbearing women with respect	64 (40.5%)	60 (70.1%)	61 (75.0%)
	Staff who rated their facility above 3 on a scale of 1 to 5 (5 being best) for care with respect and dignity they provide to women.	151 (95.6%)	86 (100.0%)	77 (95.1%)
Identification and response to Abuse	Process exist for identifying and reporting abuses during maternity and newborn care	46 (29.1%)	58 (67.8%)	41 (50.0%)
	Have encountered abuse victims in their maternity and newborn care practice	42 (26.6%)	28 (32.2%)	16 (19.8%)
	Staff who knew of 2 or more measures being implemented in their facilities to prevent abuses	31 (19.6%)	54 (62.7%)	57 (70.4%)
	Staff who mentioned 2 or more measures being implemented in their facilities to treat or rehabilitate victims of abuses	16 (10.1%)	17 (19.5%)	56 (69.0%)
	Community stakeholders' involvement in addressing disrespect and abuse of women during childbirth	49 (31.0%)	49 (57.5%)	27 (33.3%)
Payment for services	Women are expected to pay for normal deliveries	32 (20.3%)	7 (8.1%)	42 (51.9%)
	Women are expected to buy supplies for normal deliveries	132 (83.5%)	73 (85.1%)	61 (75.0%)
	Women are expected to pay before treatment in case of obstetric emergencies	19 (12.0%)	9 (10.3%)	27 (33.3%)

For peer review only

Table 3. Women's report of abuses, provider attitudes and overall satisfaction with care provided

Aspect of care	Modality for which women's perspective around facility experiences was assessed	Number of responses (%)		
		Bangladesh N=295	Ghana N=381	Tanzania N=173
Abuses during care-giving	Physical abuse from providers at the facility	6 (2.0%)	9 (2.4%)	2 (1.2%)
	Verbal abuse from providers at the facility	19 (6.4%)	25 (6.6%)	8 (4.6%)
	Sexual abuse from providers at the facility	2 (0.6%)	2 (0.7%)	1 (0.6%)
Provider relationship with caregivers	Their concerns were considered by providers in caring for them	224 (75.9%)	352 (92.4%)	108 (62.4%)
	Providers were responsive when asked for support	262 (88.8%)	366 (96.1%)	161 (93.1%)
	They were treated with respect by providers	266 (90.2%)	367 (96.1%)	168 (97.1%)
Satisfaction with care	Satisfied with attitude of providers	271 (91.7%)	353 (92.7%)	161 (93.1%)
	Satisfied with information received from providers on breastfeeding	191 (64.8%)	338 (88.7%)	132 (76.3%)
	Satisfied with information received from providers on family planning	38 (12.9%)	208 (54.6%)	77 (44.5%)

Table 4. Assessor observations of the provider attitudes during maternity care for women

Aspect of care	Modalities of care that were observed by assessors	Number of responses (%)		
		Bangladesh N=387	Ghana N=134	Tanzania N=120
Client requests	Women who made request for anything	14 (3.7%)	4 (3.0%)	4 (3.3%)
	- Those whose requests were refused with respect	1 (6.7%)	4 (100.0%)	3 (75.0%)
	Women who requested for a birth companion	286 (73.9%)	0 (0.0%)	2 (1.7%)
Payments at facility and effect on care	Women/families paid monies for some services	164 (42.4%)	19 (14.2%)	61 (50.8%)
	Women/families who complained about the payment	45 (11.6%)	1 (0.8%)	22 (18.3%)
	Women for whom services were withheld due to inability to pay	2 (0.5%)	0 (0.0%)	13 (10.8%)

For peer review only

Legend of figures

Figure 1. Availability of client toilets with soap and water for handwashing and its cleanliness

Figure 2. Women's reported experience of care at maternity units

Figure 3a-3d. Assessors' observations on women's maternity care experiences during provider interactions in health facilities

Figure 4. Mothers' satisfaction with care received in the health facilities during childbirth in the 3 countries

For peer review only

For peer review only

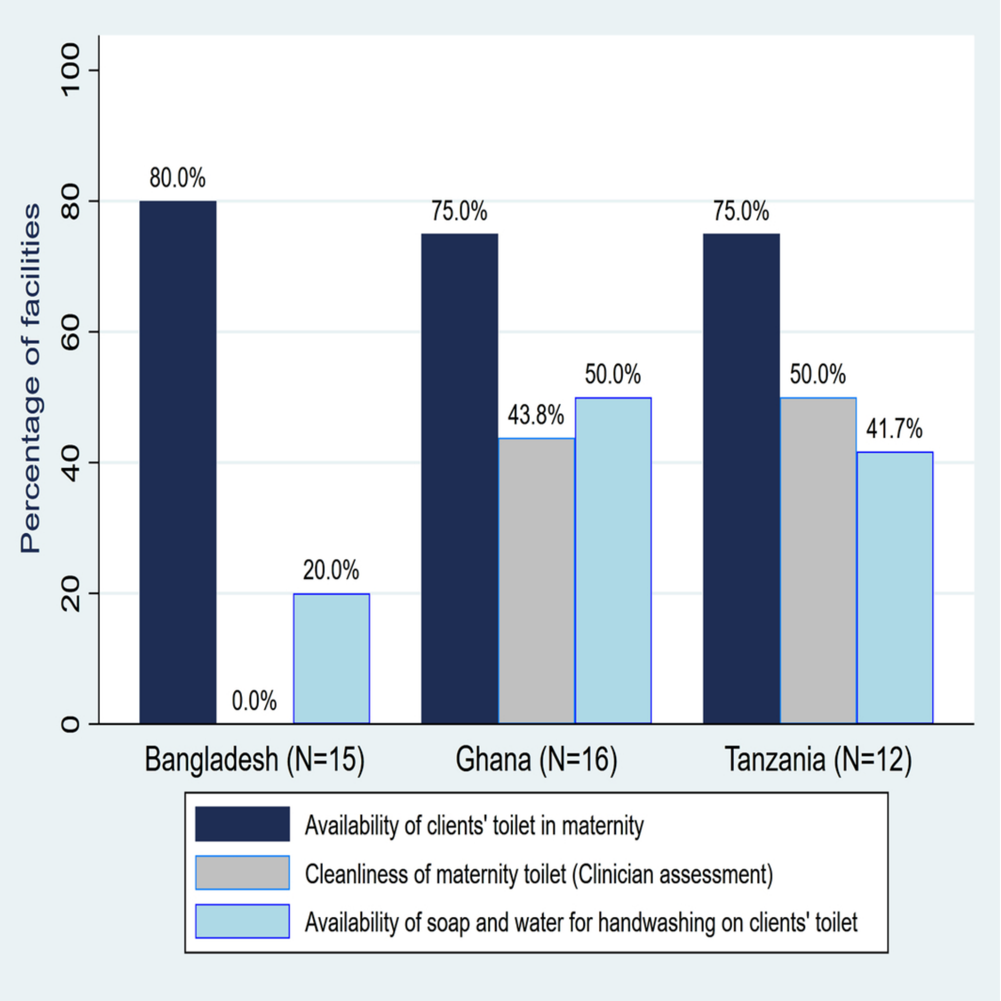

Figure 1. Availability of client toilets with soap and water for handwashing and its cleanliness

90x90mm (300 x 300 DPI)

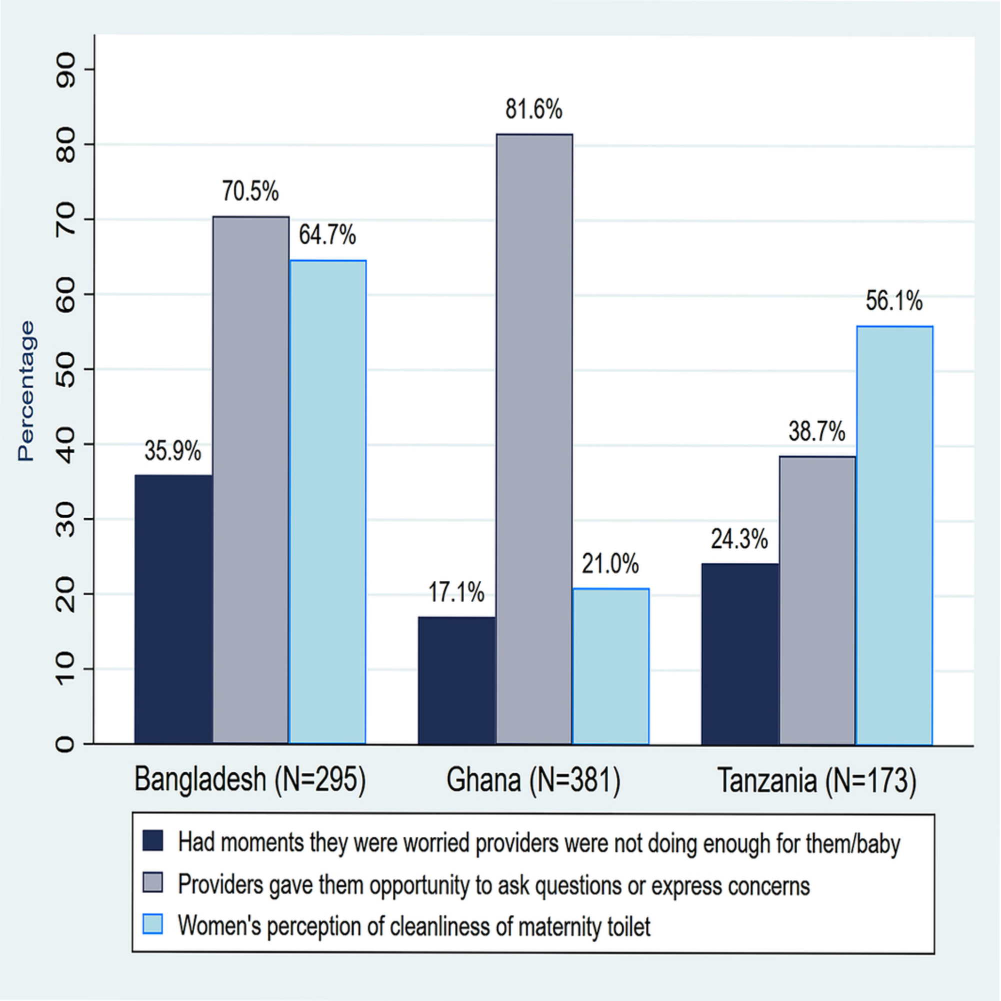

Figure 2. Women's reported experience of care at maternity units

90x90mm (300 x 300 DPI)

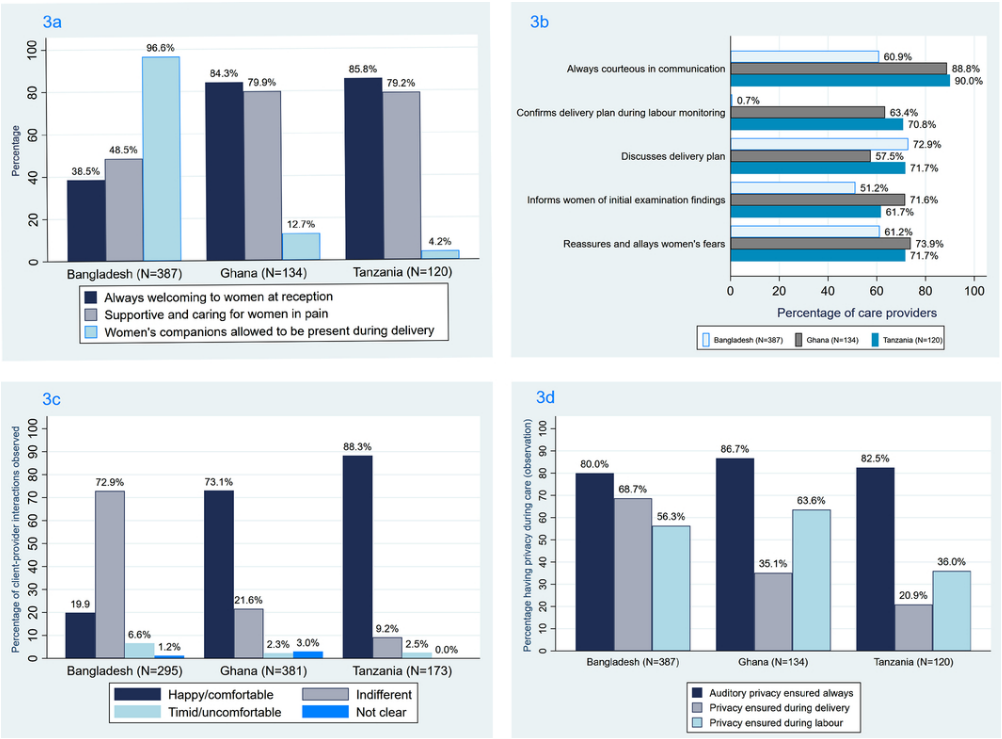

Figure 3a-3d. Assessors' observations on women's maternity care experiences during provider interactions in health facilities

90x66mm (300 x 300 DPI)

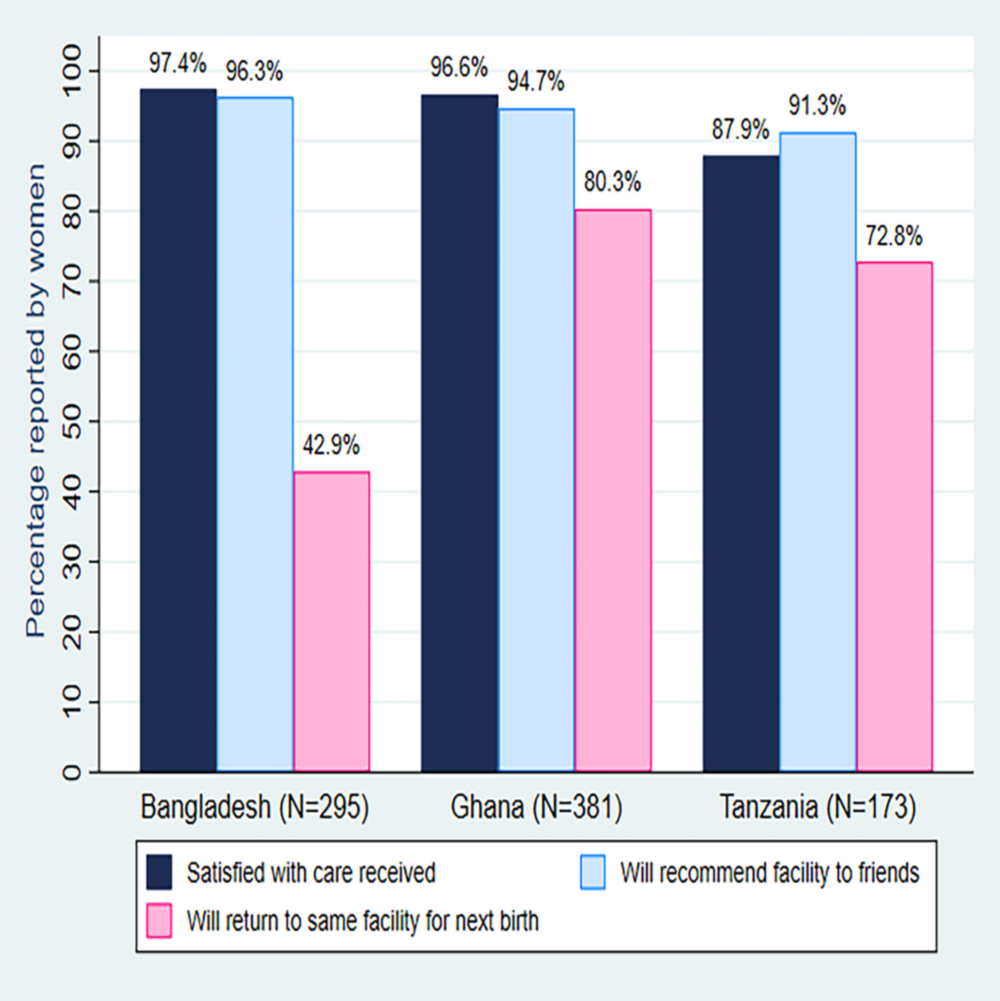

Figure 4. Mothers' satisfaction with care received in the health facilities during childbirth in the 3 countries

90x90mm (300 x 300 DPI)

STROBE guideline checklist

STROBE guidelines checklist	Page number
Title	1
Abstract	2
**Introduction**	**4**
Objectives	4
**Methods**	
Study design	4
Setting	5
Participants	5
Variables	6
Data sources / measurement	6
Bias	6
Study size	6
Quantitative variables	6
Statistical methods	7
**Results**	
Participants	8
Descriptive data	8
Outcome data	8-11
Main results	8-11
Other analysis	Not applicable
**Discussion**	
Key results	11
Limitations	12
Interpretation	11-12
Generalizability	12
**Other information**	
Funding	13

For peer review only

BMJ Open

Respectful Maternity Care delivered within health facilities in Bangladesh, Ghana and Tanzania - A cross-sectional assessment preceding a quality improvement intervention

Journal:	*BMJ Open*
Manuscript ID	bmjopen-2020-039616.R1
Article Type:	Original research
Date Submitted by the Author:	19-Aug-2020
Complete List of Authors:	Manu, Alexander; University of Ghana College of Health Sciences, Epidemiology and Disease Control/School of Public Health; Liverpool School of Tropical Medicine, Centre for Maternal and Newborn Health<br>Zaka, Nabila; UNICEF<br>Bianchessi, Christina; UNICEF,<br>Maswanya, Edward; NIMR<br>Williams, John; Navrongo Health Research Centre<br>Arifeen, Shams E.; ICDDR B, Maternal and Child Health Division (MCHD)
**Primary Subject Heading**:	Global health
Secondary Subject Heading:	Epidemiology, Health policy, Health services research, Patient-centred medicine
Keywords:	Quality in health care < HEALTH SERVICES ADMINISTRATION & MANAGEMENT, PUBLIC HEALTH, Maternal medicine < OBSTETRICS, Health policy < HEALTH SERVICES ADMINISTRATION & MANAGEMENT

SCHOLARONE™
Manuscripts

I, the Submitting Author has the right to grant and does grant on behalf of all authors of the Work (as defined in the below author licence), an exclusive licence and/or a non-exclusive licence for contributions from authors who are: i) UK Crown employees; ii) where BMJ has agreed a CC-BY licence shall apply, and/or iii) in accordance with the terms applicable for US Federal Government officers or employees acting as part of their official duties; on a worldwide, perpetual, irrevocable, royalty-free basis to BMJ Publishing Group Ltd ("BMJ") its licensees and where the relevant Journal is co-owned by BMJ to the co-owners of the Journal, to publish the Work in this journal and any other BMJ products and to exploit all rights, as set out in our licence.

The Submitting Author accepts and understands that any supply made under these terms is made by BMJ to the Submitting Author unless you are acting as an employee on behalf of your employer or a postgraduate student of an affiliated institution which is paying any applicable article publishing charge ("APC") for Open Access articles. Where the Submitting Author wishes to make the Work available on an Open Access basis (and intends to pay the relevant APC), the terms of reuse of such Open Access shall be governed by a Creative Commons licence – details of these licences and which Creative Commons licence will apply to this Work are set out in our licence referred to above.

Other than as permitted in any relevant BMJ Author's Self Archiving Policies, I confirm this Work has not been accepted for publication elsewhere, is not being considered for publication elsewhere and does not duplicate material already published. I confirm all authors consent to publication of this Work and authorise the granting of this licence.

For peer review only

Respectful Maternity Care delivered within health facilities in Bangladesh, Ghana and Tanzania - A cross-sectional assessment preceding a quality improvement intervention

Alexander Manu[1,2], Nabila Zaka[3], Cristina Bianchessi[3], Edward Maswanya[4], John Williams[5] and Shams El Arifeen[6]

[1]Centre for Maternal and Newborn Health, Liverpool School of Tropical Medicine, Liverpool, United Kingdom
[2]School of Public Health, University of Ghana, Legon, Accra
[3]United Nations Children's Fund/HQ, New York, United States of America
[4]National Institute for Medical Research, Dar es Salaam, Tanzania
[5]Navrongo Health Research Centre, Navrongo, Ghana
[6]International Centre for Diarrhoeal Diseases Research, Bangladesh, Dhaka, Bangladesh

For peer review only

Address of authors:

NZ – nzaka@unicef.org
AM – alexander.manu@lshtm.ac.uk
CB - c.bianchessi@gmail.com
EM - edward19631@hotmail.com
JW - John.Williams@navrongo-hrc.org
SA - Shams@icddrb.org

‡Correspondence to:
Dr Alexander Manu
Liverpool School of Tropical Medicine
Pembroke Place, Liverpool.
L3 5QA. United Kingdom
Email: makmanu128@gmail.com

Keywords: Respectful care, Dignified care, Quality, Maternal. Newborn, Health facility.

Word Count: 4988

ABSTRACT

Objective: To assess respectful maternity care (RMC) in health facilities.

Design:Cross-sectional study

Setting: Forty-three(43) facilities across 15 districts in Bangladesh(15), Ghana(16) and Tanzania(12).

Participants:Facility Managers; 325 providers (nurses/midwives/doctors)-Bangladesh(158), Ghana(86) and Tanzania(81) and 849 recently-delivered women-Bangladesh(295), Ghana(381) and Tanzania(173)-were interviewed. Observation of 641 client-provider interactions was conducted–Bangladesh(387), Ghana(134) and Tanzania(120).

Assessment:Trained social-scientists and clinicians assessed infrastructure, policies, provision and women's experiences of RMC(emotional support, respectful care and communication).

Primary outcome:RMC provided and/or experienced by women.

Results:Three(20%) facilities in Bangladesh, four(25%) in Ghana and three(25%) in Tanzania had no maternity clients' toilets and one-half had no handwashing facilities. Policies for RMC such as identification of client abuses were available: 81%(Ghana), 73%(Bangladesh) and 50%(Tanzania) but response was poor. Ninety-four(60%) Bangladeshi, 26(30%) Ghanaian and 20(25%) Tanzanian providers were not RMC-trained. They provided emotional support during labour care to 107(80%) women in Ghana, 95(79%) in Tanzania and 188(48.5%) in Bangladesh and were often courteous with them-236(61%) in Bangladesh, 119(89%) in Ghana and 108(90%) in Tanzania. Due to structural challenges 169(44%) women in Bangladesh, 49(36%) in Ghana and 77(64%) in Tanzania had no privacy during labour. Care was refused to 13(11%) Tanzanian and two Bangladeshi women who could not pay illegal charges. Twenty-five(7%) women in Ghana, nine(6%) in Bangladesh and eight(5%) in Tanzania were verbally abused during care. Providers in all countries highly-rated their care provision (95%-100%) and 287(97%) of Bangladeshi women, 368(97%) Ghanaians and 152(88%) Tanzanians reported "satisfaction" with the care they received. However, based on their facility experiences, significant($p<0.001$) percentages-20%(Ghana) to 57%(Bangladesh)-will not return to the same facilities for future childbirth.

Conclusions:Facilities in Bangladesh, Ghana and Tanzania have foundational systems that facilitate RMC. Structural inadequacies and policy gaps pose challenges. Many women were, however, unwilling to return to the same facilities for future deliveries although they (and providers) highly rated these facilities.

For peer review only

Article Summary

Strengths and limitations of this study

- The first key strength of this study is that it used harmonised methods and developed common tools/instruments to assess respectful maternity care in the 43 health facilities across the three countries. This makes it one of the largest multi-country facility assessments from which data is amenable to direct cross-country comparisons as well as context-relevant conclusions.

- Secondly, the study employed a variety of approaches including direct observations, interviews and records review, to assess respectful care provided in the past and what is being provided at present. Whilst observations of care providers may result in the Hawthorne effect, the use of the multiple approaches and triangulation of the findings increased the objectivity. It also improved understanding of how prevailing practices measure up to the standards at individual, structural and policy levels according to Freedman et al's framework.

- We had limitations: the cross-sectional design and the short duration of contact with facilities allowed for assessment of limited aspects of respectful care. However, it was the most pragmatic design for many settings if data collection should not unduly interrupt caregiving.

- Also, women's reported experiences and caregiver's practices may introduce respondent biases. Observing provider-client interactions may likely find improved quality of care than what is routinely provided to clientele (Hawthorne effect). Consequently, inadequacies identified during the observations may under-represent the true extent of sub-optimal provision of respectful care.

- The findings may have limited external validity due to UNICEF's equity focus in selecting areas of work in the respective countries. Most, but not all, of the assessed facilities were in the most under-served districts and this may represent a selection bias in the context of the country.

For peer review only

INTRODUCTION

In 2015, World Health Organization (WHO) issued a statement calling for global actions to prevent and eliminate disrespect and abuse during facility-based childbirth and emphasized women's right to dignified and respectful care throughout pregnancy and childbirth[1]. Facility birth rates have increased due to increased awareness, demand and access to maternity services[2]. Approximately 6.5 million more women delivered with skilled providers in 2015 and, across 60 Countdown to 2030 countries, the median skilled attendance rate increased from 65% in 2015 to 77% in 2017[3]. However, optimism around the increasing trend in facility deliveries must be cautious because deaths among pregnant women and their babies (born and unborn) have declined at a much slower pace, confirming that, unless coupled with care of the requisite quality, outcomes for women and their babies may be poor[4-8] and communities may lose trust in facilities[9 10].

D'Oliveira et al[11] and other authors found that pregnant women were abused within health facilities during childbirth, the care they are provided is not respectful or dignified[11-13]and places no value on their expectations. The WHO framework of 2016 therefore defined eight standards for improving the quality of maternal and newborn care (MNC) in health facilities along two domains – provision and experience of care[14]. It recognises the need to amplify the voices of women from low- and middle-income settings (LMICs) in the design, content and delivery of the care they receive around childbirth to ensure that it respects their dignity, preferences and aspirations[15]. The eight WHO standards were not systematically tested in any LMIC. Moreover, it is not fully described how respect and dignity of care can be objectively measured.

As part of a Mother and Baby Friendly Hospital Initiative (MBFHI), UNICEF and partners in the Ministries of Health of Bangladesh, Ghana and Tanzania are working within selected high-mortality districts to effectively improve breastfeeding and MNC outcomes. The partnership is using this platform to implement a quasi-experimental pre-post study to test the feasibility of implementing the WHO standards within these LMICs and to answer these critical questions: how pervasive is disrespectful and abusive care within health facilities in LMICs? Do facilities have and enforce conducive policy environments and/or guidelines that enable provision and experience of respectful maternity care (RMC) and what are (and can we rely on) women's perspectives on the dignity around the care they experience? Tools such as the Mothers on Respect index have not been tested in LMICs[16].

Our objective was to measure RMC from provider perspectives and as experienced by clients to labour wards in the three countries. We also aimed to assess the implications of these on women's satisfaction with the care and interpretations of how it is measured. The manuscript therefore presents findings from a baseline assessment describing existing provisions, health worker delivery and families' perceptions of RMC prior to the implementation of the standards. The results will contribute to the continuing global discussions around how to effectively measure respect and dignity in the provision and experience of maternity care.

METHODS

Study Design and Setting

For peer review only

This is a cross-sectional study, nested within the quasi-experimental pre-post evaluation of the Every Mother Every Newborn Quality Improvement (EMEN-QI) intervention. The protocol for the evaluation of the EMEN-QI intervention has been published[17]. This study had four components: inventory to check the availability of clean toilet and handwashing facilities, policies, protocols and guidelines for providing RMC; questionnaire and qualitative interviews with facility staff and maternity clients; structured observation of maternity care provided to clients and review of client medical records for documented care.

It was carried out by researchers from three research institutions from the three countries– International Center for Diarrhoeal Diseases Research in Bangladesh (ICDDR,B), Navrongo Health Research Centre (NHRC), Ghana Health Service and the National Institute for Medical Research (NIMR), Tanzania. It was conducted between May and August 2016 in 43 health facilities in Bangladesh (15), Ghana (16) and Tanzania (12), comprising 1 regional and 12 district hospitals and 30 health centres (HCs)/upazilla health complexes (UHCs). Teams of 2-4 clinicians (CRAs) and research social scientists/anthropologists (RSAs) carried out the assessment in each facility. These teams were trained by the principal investigators in each country under the direct supervision of the lead author. The training was followed by pretesting exercises which included hospital visits until there was consensus on the observations made for client-provider interactions.

In Bangladesh, three hospitals and twelve UHCs in Kurigram, Lalmonirhat and Gaibandha districts of the Rangpur division were involved. In Ghana, the regional hospital of the Upper East Region (UER), five district hospitals and ten health centres in eight districts (Bawku, Kassena-Nankana East and Bolgatanga Municipalities and Bawku West, Bongo, Kassena-Nankana West and Builsa North Districts) were covered. For Tanzania, the assessments were done in four hospitals and eight health centres within Njombe Town Council, Makete, Ludewa and Wanging'ombe districts of the Njombe region. These facilities provide basic/comprehensive emergency obstetric care (BEmOC/CEmOC) services and were selected in consultation with the ministries of health of the respective countries to reflect high average caseload, relatively poor maternal and newborn health indices and nationally-representative human resource availability.[17] All district hospitals, UHCs in Bangladesh and one HC Tanzania provided CEmOC services.

Participants

To cover provision and experience of care, respondents for the study included facility managers and "in-charges", providers who directly cared for women in labour for the two weeks of the assessment, recently-delivered women and their companions. The health records of all women who were seen and cared for at the maternity in the 3-6 months prior to the assessment were reviewed and data abstracted.

Informed Consent

We consented all participants to participate in the study. Research Assistants read out or gave an information sheet to all participants explaining the purpose of the assessment in a language of their choice. Each participant asked for clarifications on the study. They were informed that there will be no direct benefits for participation but the information they provide will help improve the quality of maternity care provided in facilities. They were assured of their right to withdraw participation at any time without affecting their position in the facility or care they received and that information provided

For peer review only

will be treated as confidential. Agreement to participate was indicated with a signature/thumbprint. No respondent refused participation in the study. All interviews lasted 45-60 minutes.

Ethics Approval

The study protocol was approved by institutional ethics review boards (IRBs) in all three countries: ICDDR,B in Bangladesh (PR-16024, 1st June 2016), Ghana Health Service (GHS) in Ghana (NHRCIRB226, 6th April 2016) and NIMR in Tanzania (NIMR/HQ/R.8a/Vol. IX/2176, 11th April 2016). Clearance was sought from the facility managers for the study in the respective facilities.

Patient and Public Involvement

The study tools and instruments were pretested in all the three countries to solicit facility-user input into the strategy for data collection. The data collection involved patients or maternity clients who have been discharged from the facilities as well as their companions who accompanied them to the facility for the delivery. Their identities were protected, and their confidentiality and privacy were ensured all the information they provided. Their contributions have been cited in the acknowledgements.

Assessment of Respectful Maternity Care

Measurement of Respectful maternity care (RMC): RMC was measured along the dimensions of the three components in the WHO framework - emotional support, respectful communication and respectful and dignified care - from both the perspectives of the provider, the women (clients) and an independent informed assessor (research clinicians or CRAs). These were supplemented with data on the availability of the right policy environment and facility provisions (e.g. handwashing) for RMC.

After facility entry, CRAs interviewed 43 facility superintendents/"in-charges" and assessed whether the facility had specific protocols and guidelines for ensuring RMC including staff training at maternity units. Using a semi-structured questionnaire, the CRAs assessed whether facilities had systems for identifying abuses of pregnant women seeking or receiving care including "whistle blower" policies where care providers could report client abuses perpetrated by their peers under protected or concealed identities. Respondents were asked how they involved communities in defining and providing quality and woman-centred maternity care. We considered the availability of clean client toilets on the maternity as a basic necessity in providing respectful care and so CRAs checked whether maternity wards had toilets and rated their cleanliness based on agreed criteria.

CRAs also 325 interviewed care providers in the maternity wards on any formal RMC training they received, any encounter with situations of client abuse in facilities and management responses to abuses. Care providers included specialists, doctors, midwives and staff nurses. Selection was biased towards staff who play key leadership or decision-making roles in facilities (e.g. matrons who provide care and enforced guidelines and regulations) or served for more than six months.

The CRAs also passively 641 observed client-provider interactions as women navigated through all the points of care provision within facilities–from reception through till discharge after delivery. This provided first-hand experience of auditory and visual privacy and provider-client communication including explanation of the care plan and the involvement of clients in their own care. They checked whether providers ensured a conducive atmosphere for women and their companions to ask

questions on the care they received; support (emotional and other) for women during childbirth; and any abuses. They reviewed women's records to assess documentation of care.

RSAs identified isolated, comfortable and conducive spaces within facility to interview 829 recently-delivered women who have been discharged from facilities on their experience of maternity care provided. All women who have been in the facility for at least six hours and consent to participate were interviewed irrespective of birth outcomes. This was because women who present in labour are observed for at least 6 hours after the birth before discharge, creating opportunity for them to interact with providers. The RSAs ensured that respondents were not seen or heard during the interview. The interview covered women's perspectives on provider communications, responsiveness, respect for their choices and preferences, visual and auditory privacy during care provision, demand for informal or formal payments and withholding of care if such demands were refused, any physical, sexual or verbal abuses during women's stay in the facility. Clients' impressions and satisfaction with the maternity care they experienced were also elicited but aside that, they were asked whether their experiences will make them recommend the facility to relatives or friends for maternity care or whether they themselves will like to return to the same facility for their next childbirth.

RSAs also interviewed any persons accompanying the women during their stay in the facility for their independent perspectives on the care provided to their relative or friend. RSAs asked whether providers demanded or collected payments for services or whether any service was withheld for any reason including inability to pay.

Quality assurance and data processing

Stringent uniform quality assurance (QA) processes were implemented starting from the field through to the data processing units of the research institutions. Team coordinators liaised between fieldworkers, facility leadership and data centres. Data collection was paper-based in Ghana and Bangladesh but electronic in Tanzania. At the end of each day, research teams met and peer-reviewed each other's forms for completeness. Team coordinators re-checked all forms to ensure blanks or inconsistencies were identified and resolved. All checked forms were batched, logged into batch control sheets and transmitted to computer centres for processing.

In Ghana and Bangladesh, two data entry clerks independently entered the data and the two data streams were compared for agreement. Any disagreements were resolved with the source questionnaire by data managers who then run range and consistency and data integrity checks to generate exception reports for the study investigators to review and resolve. In Tanzania, data were directly transmitted to central servers for review by data managers. Where required, forms with challenges were sent back to the field for resolution. Cleaned data were securely stored on password-protected servers. A copy of the final data was shared with UNICEF/HQ.

Data analyses

Data were transferred to Stata version 14.1 for analyses. Data were represented with tabular, numerical and graphical methods. Proportions, means(sd) and medians (IQR) were estimated for the outcome analyses. Z-tests were performed to assess differences in proportions and χ^2 tests for association between variables. All tests were significant at the 5% level and 95% confidence intervals were constructed around point estimates where necessary. Responses to open-ended questions were

For peer review only

coded into themes in NVIVO 12.0 after repeated readings using the framework approach. Qualitative analyses involved exploration of relationships between themes and were triangulated to the close-ended questions to facilitate their contextual interpretations.

Coordination

UNICEF coordinated the uniform assessment across countries. A consultant (AM) facilitated a UNICEF-organised design meeting and training in New York with principal investigators (PIs). Skype conference calls were organized every week to agree harmonised timelines for implementation and update on progress. The coordinated approach allowed for common solutions to challenges encountered and for the consultant to visit each country during the training and initiation of data collection.

RESULTS

Health facility managers in all 43 facilities across the three countries were interviewed. There were 325 care provider interviews with nurses, midwives and doctors comprising 158 in Bangladesh, 86 in Ghana and 81 in Tanzania. Questionnaires were administered by social scientists/anthropologists to collect data from a total of 849 recently-delivered women: 295 from Bangladesh, 381 from Ghana and 173 from Tanzania. Observation of 641 client-provider interactions was conducted by CRAs – 387 in Bangladesh, 134 in Ghana and 120 in Tanzania.

Availability of dedicated clean maternity toilet as part of respectful maternity care

Clean toilets with handwashing facilities were inadequate in the maternity units in all three countries (figure 1). Three (20%) facilities in Bangladesh, four (25%) in Ghana and three (25%) in Tanzania did not have client toilets in the maternity. Where they existed, they were judged as unclean by study clinicians-6(50%) in Tanzania, 9 (56%) in Ghana and 15(100%) in Bangladesh-and without soap and water for handwashing-7 (58%) in Tanzania, 8 (50%) in Ghana and 12 (80%) in Bangladesh. Women's perceptions on the cleanliness of the toilets contrasted with these clinician observations. Figure 2 shows that only 104(35%) women interviewed in Bangladesh and 301 (79%) in Ghana thought the toilets were unclean.

Availability of the right policy environment for respectful maternity care

Majority (13 representing 81.3%) of facility managers in Ghana, compared to 11 (73.3%) in Bangladesh and 6 (50.0%) in Tanzania, reported that clear processes exist for identifying client abuses (Table 1). In Ghana, all but one facility had a "whistle-blower" policy for reporting abuses, and all facilities (100%) actively encourage women to report any provider abuses. In Tanzania only one in four facilities had a "whistle-blower" policy and one in three encourage women to report abuses. Data on whistle blower policies were not captured in Bangladesh although, in qualitative interviews, staff suggested that mechanisms for making anonymous complaints using mobile SMS exist, are captured onto the district health information systems (DHIS-2) platform and followed-up regularly.

Consistent with facility managers' reports, assessors found that, of all three countries, most health facilities in Ghana had policies on women's rights during care at the facility. Sixty-one (71%) providers in Ghanaian facilities reported that they had policies on addressing client concerns and 70 (82%) reported that policies exist on the rights of clients who sought maternity care (table 2). In Bangladesh, only 54(41%) of providers interviewed reported the existence of policies to address client concerns

For peer review only

and 83(53%) on women's rights although the staff could not show copies of the policy documents to the assessors during the visits. In Tanzania, 54 (67%) providers reported that their facilities had policies to address client concerns whilst 57 (70%) reported that policies on women's rights whilst receiving care (table 2)

Training of providers in respectful maternity care

Specific training on how to provide maternity care with dignity and respect was not provided to 30%-60% of providers across the three countries. In Bangladesh, 127 (80%) providers could not mention two measures being implemented in their facilities to prevent abuses of women during maternity care (Table 2). There were 32 (37%) providers in Ghana and 24 (30%) in Tanzania who could also not mention two or more measures. A substantial proportion-69 (80%) in Ghana and 142 (90%) in Bangladesh-did not know of two or more measures in place to treat or rehabilitate victims of abuse. Meanwhile, table 2 also shows that almost all of these providers highly rated the respect and dignity in the care they provided scoring it above 3 on a scale of 1 to 5, 5 being the best-151 (96%) of them in Bangladesh, 86 (100%) in Ghana and 77 (95%) in Tanzania.

Women's reported experience of maternity care and how it compares with assessors' observations

Whilst most women reported that they were happy with the attitude of providers (table 3), they also said the provider was not doing enough for them and/or their babies and were not given the opportunity to express these concerns (figure 2). For example, in Tanzania, although 168 (97.1%) of women reported that providers treated them with "respect" (table 3) and 161 (93.1%) said providers were responsive to them when they asked for support, figure 2 also shows that about a quarter of them 42 (24.3%) reported about moments when they were worried that the providers were not doing enough for them. Moreover, 106 (61.3%) reported that they had no opportunity to express these concerns or ask questions. The trends were similar in Ghana and Bangladesh; in Bangladesh, for instance, 266 (90.2%) women reported being treated with "respect" but 106 (35.9%) had moments they were concerned that the providers were not doing enough for them and 87 (29.5%) thought they were not given the opportunity to express these concerns.

During the observation of client-provider interactions by the study assessors varying trends were found between the three countries. Figures 3a and 3b show that in Ghana and Tanzania, at reception, the providers were mostly welcoming to 113 (84%) and 103 (86%) respectively and were supportive and caring when women were in labour pain 107 (80%) and 95 (79%) respectively. The assessors also found that the providers were "always courteous" in communication with the women-119 (89%) in Ghana and 108 (90%) in Tanzania). In the two countries, providers informed 62%-72% of women about their initial examination findings, discussed delivery plans with 52%-72% of them, reassured them and allayed their fears in over 70% of cases and confirmed delivery plans during labour monitoring in 63%-71% of cases. However, figure 3c shows that, in these same countries, 12%-27% of respondents were not "comfortable" talking to the providers and 2.3%-2.5% were visibly uncomfortable or looked timid. Only 3% of women in the two countries made specific requests from the providers and none in Ghana could request for a birth companion (Table 4).

In contrast, the findings were different in Bangladesh as uptake of these modalities in RMC were lower. Figures 3a and 3b show that providers were "always welcoming" to 149 (36%) of women, were supportive and caring during labour pains in 188 (49%) and just about half 198 (51%) were told

For peer review only

examination findings at initial exams. Though the delivery plan was discussed with 282 (73%) of the women, during labour monitoring, these plans were confirmed with only 3(0.7%) of them. Table 4 shows that about 14 (4%) of women in Bangladesh made specific request from the providers and though all were refused, it was only in a single case that the refusal was done respectfully. Figure 3c shows that 25 (7%) of women in Bangladesh felt uncomfortable and timid when talking to the providers, about three-times those in Ghana and Tanzania.

Companion at birth

No woman in Ghana and only two (2%) in Tanzania reported requesting a birth companion for the delivery (Table 4). This contrasts with the 286 (74%) in Bangladesh. However, in figure 3a, women's companions were allowed to be present for 17 (13%) of women in Ghana, 5 (4%) in Tanzania and 374 (97%) in Bangladesh.

Verbal, physical or sexual abuse of women during maternity care

Facility managers interviews (Table 1) showed that cases of abuse of women during maternity care were reported but measures to prevent recurrence were weak. Managers were aware of recent (less than 2 weeks) cases of abuse in their facilities. Five cases of abuse were reported in Ghana compared to only one each in Bangladesh and Tanzania. Staff also reported encounters with victims of abuse in their practice. About 16 (20%) providers in Tanzania, 42 (27%) in Bangladesh and 27 (32%) in Ghana had encountered victims of abuse in their practice but up to 70% of providers between the three countries did not know of any existing systematic processes for identifying and reporting abuses within their facilities. Meanwhile, more women reported that they were physically, verbally or sexually abused by providers during care (Table 3). The commonest form of abuse was verbal; on average, 6% of women in all three countries experienced this form of abuse (5% in Tanzania, 6% in Bangladesh and 7% in Ghana). Sexual abuses were the rarest with only 1 or 2 women reporting the experience in each country. On average, 2% of the women also experienced physical abuses from providers during their stay in the facility.

When these abuses occurred, facilities in Ghana and Tanzania conducted internal investigations and the only action taken to prevent future occurrences was counselling or verbal warnings to the staff involved (5 of the 6 cases). In comparison, the only recent case in Bangladesh was referred to an external body for investigation and action.

Privacy of care

During assessors' observations of client-provider interactions, of the three countries, auditory privacy was ensured in over 80% of interactions (Figure 3d). Privacy during labour was ensured mostly in Ghana where in 64% of the 134 client-provider interactions observed during labour, privacy was ensured. In Bangladesh, privacy in labour was ensured for 218 (56%) of interactions and it was for 43 (36%) in Tanzania . During delivery care was least ensured in Tanzania (21% of 120 observations). It was 35% in Ghana and 69% in Bangladesh.

Demand for payment and withholding of delivery care services for inability to pay

Consistent with findings from CRA observations of client-provider interactions, providers in all three countries reported that women were made to pay monies for supplies and/or delivery, even in

obstetric emergencies (table 2). Charging fees was most common within facilities in Tanzania where 42 (52%) providers reported that women are made to pay fees for normal deliveries. Twenty-seven (33%) providers in Tanzania also said these payments were also expected for obstetric emergencies. The proportion of providers reporting these payments were relatively lower in Bangladesh where 32 (20%) reported payments for normal deliveries and 19 (12%) in emergencies. In Ghana, where a National Health Insurance Scheme covers the cost of facility births, 7 (8%) providers reported that some illegal fees were still charged for normal deliveries and 9 (10%) said there were fees charged for obstetric emergencies. Seventy-three (85%) providers in Ghana, 132 (83%) in Bangladesh to 61 (75%) in Tanzania reported that women had to buy supplies for normal deliveries in the facility.

During observation of client-provider interactions, study assessors found that providers demanded monies from women for maternity services. This practice was most common in Tanzania where 61 (51%) cases observed, followed by 164 (42%) in Bangladesh and 19 (14%) in Ghana. Maternity services were sometimes withheld from women when they are unable to pay and, whilst some complained about the payments in general, they were in the minority. During observations, one mother in Ghana, 45 (12%) in Bangladesh and 22 (18%) in Tanzania were seen complaining about the charges. It is noteworthy that some delivery care services were withheld from women (11% in Tanzania and 0.5% in Bangladesh) because inability to pay. No such service was withheld in Ghana.

Community involvement providing respectful maternity care

Managers of facilities reported that six (50%) facilities in Tanzania, eight (53%) in Bangladesh twelve (75%) in Ghana involved community stakeholders in deliberations around ensuring respectful maternity care (table 2). Facility providers corroborated this. When care was abusive or not respectful, 49 (31%) providers in Bangladesh, 27 (33%) in Tanzania and 49 (58%) reported that community stakeholders were involved in addressing it.

Overall satisfaction with the maternity care at health facilities

On a scale of 1 to 5 (5 being the best) in terms of RMC provided to women, all (100%)providers from Ghana, 151 (96%) from Bangladesh and 77 (95%) from Tanzania ranked their facilities 3 and above. Over 90% of women from all countries also reported satisfaction with the attitude of the providers - 271 (91.7%) in Bangladesh, 353 (92.7%) in Ghana and 161 (93.1%) in Tanzania (Table 3). Figure 4, however, shows that majority of women expressed satisfaction with the maternity care they received at facilities-287 (97%) in Bangladesh but, on the contrary, only 106 (43%) will to return to the same facilities for future maternity care and this difference was statistically significant ($p<0.001$). Similarly, 386 (97%) in Ghana expressed satisfaction with the care but only 305 (80%) will return to the same facility whilst in Tanzania, 152 (88%) were satisfied but and only 126 (73%) will return to the same facilities in the future for maternity care; differences that were statistically significant ($p<0.0001$). Meanwhile many of them reported that they would recommend the facilities to a relative or friend for maternity care even if they themselves will not do same in the future-96% in Bangladesh, 95% in Ghana and 91% in Tanzania.

DISCUSSION

Though respectful maternity care is a relatively new and developing concept in many LMICs, our assessment in the three countries found that infrastructure, policies on caregiving and staff readiness

For peer review only

for providing RMC were in place to build upon. Three-quarters and more of the facilities had separate toilets in the maternity for women and half to these had handwashing facilities. Providers and managers' reports showed that approximately seven out of ten facilities in Ghana and Tanzania had policies on patients' rights and procedures for addressing their concerns but only in 40%-50% in Bangladesh. Prior to implementing EMEN-QI, uptake of practices around the three RMC domains (effective communication, supportive care and dignified and respectful care) is variable across the three countries but generally high. Without this right policy environment, women did not have warm reception, examination findings were not communicated, women's concerns were not considered in the care they received and they were not supported during labour. Structural inadequacies did not allow for privacy during maternity care for women and birth companions were also not present especially in Tanzania and Ghana. Moreover, illegal payments were demand from women for delivery care and services were sometimes withheld due to inability to pay. With the right processes for ensuring patient rights including "whistle blowers" policy, as in Ghana, abuse reporting was higher. Facility providers over-rated RMC they provided as compared to what was observed. Between 50% to 75% of facilities already involve community stakeholders in addressing RMC experiences of women. Although most women across the three countries reported "satisfaction" with care provided at facilities, they will not come back to the same facilities for their next delivery based on their experiences.

Our findings align with those from earlier studies suggesting that measuring satisfaction with care with questions that merely ask whether women or providers were satisfied with care received or provided was unreliable[9]. Our results show that whilst 88%-97% of women and providers highly rated their satisfaction with care received or provided, perhaps a probable indicator of satisfaction with care may be the significant 20% to 58% (p<0.0001) of women who reported that, based on the maternity care experienced, they will not return to the same facilities for birth in the future. This apparent inconsistency between women's reported satisfaction with care and desire to return to same facility has not often been measured although alluded to when researchers find unbelievably high reported level of satisfaction under situations that did not appear to merit same[9 10]. Lack of respectful and dignified care are known barriers to subsequent careseeking[18]. This indicator will require testing in robust studies.

There appeared differences in observed provider attitudes between the South Asian (SA) and sub-Saharan African (SSA) facilities in that, apart from Bangladesh, Figure 3a shows that providers were mostly welcoming to women and supported them during labour pains. With only 39% of providers welcoming women and 48% supporting in Bangladesh compared to over 80% Ghana or Tanzania, it was not surprising that about 7% of women were intimidated to talk to providers. Meanwhile, 17%-36% of women across the three countries were sometimes concerned that the providers were not providing enough care for them but could not express. The UK quality care commission[19] defines respect and dignity in care as care "that treats care recipients as equals" who merely require support from care providers to be autonomous, independent[20]. This definition conforms with 4th, 5th and 6th of the WHO standards for facility quality of care for mothers and newborns[14].

Abuse is a seen as a way of controlling clients to undermine their autonomy[11]. The assessment findings confirm that, across the three countries, physical, sexual, verbal and psychological abuses (where services are withheld from women because of inability to pay informal/illegal charges) were experienced by women. These findings concur with reports of care providers "scolding" or "shouting

For peer review only

at" women and verbalising words that were "unkind, brusque, rude, unsympathetic and uncaring" to them[21 22]. We believe that the 4.6%-6.6% of verbal abuses may represent gross under-reporting and that challenge might be bigger. Also, providing RMC requires development of infrastructure. For instance, none of the three countries prohibits the use of birth companions but the set-up of the units did not allow for privacy or birth companions in these units.

Respectful care is also not merely the absence of abuses. Policies on the rights of maternity clients and for addressing abuse are not always present in facilities particularly in Tanzania. Even when they existed, providers were oblivious, or the enforcement was weak. A conducive policy atmosphere facilitates reporting of abuse cases. Our finding that 25% (Tanzania) to 60% (Bangladesh) of providers have not been trained on provision of dignified and RMC is unfortunate. For instance, Ghana reported the most advanced policy environment to identify and address abuses including a "whistle-blower" policy. Consequently, women in Ghana were the most likely to report of abuses. This suggests that addressing abuses in maternity care requires deliberate efforts to create conducive environments that address systems rather than individual failures that occasion "blame" – literally "A systems approach in a person-centred system"[23 24].

The WHO statement affirms every woman's right to dignified, respectful healthcare, identifying an increasing need for more research to measure the burden and pervasiveness of disrespect and abuse in health facilities[1 21 22 25]. Vogel et al[25] added that poor measurement of respectful care may result from the lack of globally applicable terminologies. Initiatives to improve respect around childbirth care are not new in these countries albeit on smaller scales: Bangladesh has implemented a Women Friendly Health Services Initiative in some facilities but Kurigram district did not benefit. Similarly, the Ifakara Research Institute in Tanzania is also implementing RMC interventions in some facilities. These have not permeated the healthcare systems. That quality affects outcomes is incontrovertible[26]. We believe that efforts to provide RMC must be intentional, goal-directed, measurable, backed by a strong political will, and should have robust systems of accountability[27].

This study has many strengths: first, using harmonised methods to assess the 43 facilities makes it one of the largest multi-country facility assessments from which data is amenable to direct cross-country comparisons. Secondly, the use of a variety of approaches including observations and data review increased the objectivity and improved understanding of how prevailing practices measure up to the standards at individual, structural and policy levels according to Freedman et al's framework[28].

We had limitations: the cross-sectional design does not allow for attribution of causality and the short duration of contact with facilities limited the aspects of respectful care that was measured. However, it was the most pragmatic design for many settings if it should not unduly interrupt caregiving. Women's reported experiences and caregiver's practices were subject to biases. Observing provider-client interactions may likely to make them go beyond what they usually do (Hawthorne effect). Consequently, inadequacies identified during the observations may represent a small dip in an abyss of inadequate respect in care. These findings may not be generalisable to all facilities in the respective countries because, due to UNICEF's equity focus, assessed facilities were located in most under-served and least-developed districts which may imply selection biases. The assessment did not measure the psychometric properties of any of the indicators and merely generated proposed candidate indicators for future testing.

For peer review only

This study contributes to amplifying women's voices, which is key to the values of the WHO/UNICEF-coordinated Quality, Equity, Dignity network and the recent petition on "What women want?"[29]. However, women's aversion to care that is not dignified or respectful, nuanced in their decision not to patronise these facilities in the future should alert health systems that community trust in facilities for delivery care should not be taken for granted. The Lancet Quality Commission advocates for measurement of client satisfaction on a systematic basis[9].

In conclusion, RMC provided and consequently experienced by women across the three countries had many good aspects that could form a foundation for quality improvement interventions. Health providers' accounts over-rated the respect and dignity in the care they provide to women way beyond what was directly observed. Critical gaps still exist which a systematic approach to implementation of RMC, as envisaged in the EMEN-QI initiative, should address. It calls for more rigorous measurement of efficacy of these measures as well as in-depth anthropological investigation to understand the drivers of respectful and dignified care to help develop corrective strategies and accountability mechanisms around these in a participatory. As a first step, respectful care must form a critical component of pre-service training in all countries. Community engagement through organizing women groups for a systematic demand creation around respectful care, follow-up and inclusion in a holistic system response will be key. Periodic client-exit interviews, use of mobile Short Messaging Services, comment boxes or easy-touch buttons for client feedback will be useful but need to be coupled with capacity to retrieve these data, systematically analyse them and use them to inform changes. Health systems and facilities should be bold to empower an approachable community ombudsman to listen to client complaints and provide systematic feedback into the delivery RMC in facilities[30] . Until then, our mothers will not return to our facilities after their experiences with providers in facilities.

Author contributions

The study was designed by AM, NZ, EM, JW, and SA with technical input from CX. AM, NZ, EM, JW, SA, formed a team that managed and supported the respective countries to implement the evaluation. AM and NZ drafted the manuscript and obtained inputs from the co-authors: JW, EM, SA, CX. The revised manuscripts were proof-read by all authors who helped in finalising it. All co-authors (NZ, AM, EM, JW, SA) read the final manuscript and agreed to its submission for publication in this journal.

Acknowledgements

This work was conducted under the UNICEF-BMGF MNCH partnership. We also appreciate the contributions from Dr Priscilla Wobil (UNICEF Ghana), Masum Billah (ICDDR,B Bangladesh), Stella Kilima (NIMR, Tanzania), Francis Yeji (NHRC/GHS, Ghana), management and staff of our collaborating institutions at the International Centre for Diarrhoeal Disease Research, Bangladesh, Navrongo Research Centre of the Ghana Health Service, National Institute of Medical Research, Tanzania and Dr Tedbabe Hailegebriel, Dr Will Zeck and Seun Oyedele of UNICEF/HQ and the UNICEF country offices in the three countries. The multiple consultations with a wide range of stakeholders including global/international experts and professionals in maternal and newborn health, ministries of health

For peer review only - http://bmjopen.bmj.com/site/about/guidelines.xhtml

and partners in Bangladesh, Ghana and Tanzania. We also thank the mothers who participated in the design and provided data for this analysis.

Competing interests

All the authors declare no conflict of or competing interests.

Funding

The study was funded by the Bill and Melinda Gates Foundation through UNICEF headquarters, New York (Grant Number OPP1112117). The findings and conclusions in this report are those of the authors and do not represent the official position of these organizations.

Data Sharing Statement

The data analysed for this manuscript is part of the bigger EMEN assessment being undertaken by UNICEF. Access to the data will follow site-specific and national guidelines of the three countries and agreements with UNICEF HQ.

For peer review only

References

1. World Health Organization. The prevention and elimination of disrespect and abuse during facility-based childbirth. In: Department of Reproductive Health and Research WHO, ed. Geneva, Switzerland: World Health Organization, 2015.
2. Ng M, Misra A, Diwan V, et al. An assessment of the impact of the JSY cash transfer program on maternal mortality reduction in Madhya Pradesh, India. *Glob Health Action* 2014;7:24939. doi: 10.3402/gha.v7.24939
3. Countdown to 2030 Collaboration. Countdown to 2030: tracking progress towards universal coverage for reproductive, maternal, newborn, and child health. *Lancet* 2018;391(10129):1538-48. doi: 10.1016/S0140-6736(18)30104-1 [published Online First: 2018/02/06]
4. Kruk ME, Gage AD, Joseph NT, et al. Mortality due to low-quality health systems in the universal health coverage era: a systematic analysis of amenable deaths in 137 countries. *Lancet* 2018;392(10160):2203-12. doi: 10.1016/S0140-6736(18)31668-4 [published Online First: 2018/09/10]
5. Lawn JE, Blencowe H, Waiswa P, et al. Stillbirths: rates, risk factors, and acceleration towards 2030. *Lancet* 2016;387(10018):587-603. doi: 10.1016/S0140-6736(15)00837-5 [published Online First: 2016/01/23]
6. Ronsmans C, Graham WJ, Lancet Maternal Survival Series steering g. Maternal mortality: who, when, where, and why. *Lancet* 2006;368(9542):1189-200. doi: 10.1016/S0140-6736(06)69380-X
7. Doyle C, Lennox L, Bell D. A systematic review of evidence on the links between patient experience and clinical safety and effectiveness. *BMJ Open* 2013;3(1) doi: 10.1136/bmjopen-2012-001570 [published Online First: 2013/01/08]
8. Souza JP, Gulmezoglu AM, Vogel J, et al. Moving beyond essential interventions for reduction of maternal mortality (the WHO Multicountry Survey on Maternal and Newborn Health): a cross-sectional study. *Lancet* 2013;381(9879):1747-55. doi: 10.1016/S0140-6736(13)60686-8
9. Kruk ME, Gage AD, Arsenault C, et al. High-quality health systems in the Sustainable Development Goals era: time for a revolution. *Lancet Glob Health* 2018;6(11):e1196-e252. doi: 10.1016/S2214-109X(18)30386-3 [published Online First: 2018/09/10]
10. Larson E, Leslie HH, Kruk ME. The determinants and outcomes of good provider communication: a cross-sectional study in seven African countries. *BMJ Open* 2017;7(6):e014888. doi: 10.1136/bmjopen-2016-014888 [published Online First: 2017/07/05]
11. d'Oliveira AF, Diniz SG, Schraiber LB. Violence against women in health-care institutions: an emerging problem. *Lancet* 2002;359(9318):1681-5. doi: 10.1016/S0140-6736(02)08592-6 [published Online First: 2002/05/22]
12. Silal SP, Penn-Kekana L, Harris B, et al. Exploring inequalities in access to and use of maternal health services in South Africa. *BMC Health Serv Res* 2012;12:120. doi: 10.1186/1472-6963-12-120 [published Online First: 2012/05/23]
13. Small R, Yelland J, Lumley J, et al. Immigrant women's views about care during labor and birth: an Australian study of Vietnamese, Turkish, and Filipino women. *Birth* 2002;29(4):266-77. [published Online First: 2002/11/15]
14. World Health Organization. Standards for improving quality of maternal and newborn care in health facilities. Geneva, Switzwerland: World Health Organization, 2016.
15. de Bernis L, Kinney MV, Stones W, et al. Stillbirths: ending preventable deaths by 2030. *Lancet* 2016;387(10019):703-16. doi: 10.1016/S0140-6736(15)00954-X [published Online First: 2016/01/23]
16. Vedam S, Stoll K, Rubashkin N, et al. The Mothers on Respect (MOR) index: measuring quality, safety, and human rights in childbirth. *SSM Popul Health* 2017;3:201-10. doi: 10.1016/j.ssmph.2017.01.005 [published Online First: 2018/01/20]

For peer review only

17. Manu A, Arifeen S, Williams J, et al. Assessment of facility readiness for implementing the WHO/UNICEF standards for improving quality of maternal and newborn care in health facilities - experiences from UNICEF's implementation in three countries of South Asia and sub-Saharan Africa. *BMC Health Serv Res* 2018;18(1):531. doi: 10.1186/s12913-018-3334-0 [published Online First: 2018/07/11]
18. Bohren MA, Hunter EC, Munthe-Kaas HM, et al. Facilitators and barriers to facility-based delivery in low- and middle-income countries: a qualitative evidence synthesis. *Reprod Health* 2014;11(1):71. doi: 10.1186/1742-4755-11-71 [published Online First: 2014/09/23]
19. Care Quality Commission UK. Health and Social Care Act 2008 (Regulated Activities) Regulations 2014: Regulation 10. http://www.cqc.org.uk/guidance-providers/regulations-enforcement/regulation-10-dignity-respect.
20. World Health Organization; Organisation for Economic Co-operation and Development; and The World Bank. Delivering quality health services: a global imperative for universal health coverage. Geneva, Switzerland: World Health Organization Organisation for Economic Co-operation and Development and The World Bank., 2018.
21. Bohren MA, Vogel JP, Tuncalp O, et al. "By slapping their laps, the patient will know that you truly care for her": A qualitative study on social norms and acceptability of the mistreatment of women during childbirth in Abuja, Nigeria. *SSM Popul Health* 2016;2:640-55. doi: 10.1016/j.ssmph.2016.07.003 [published Online First: 2017/03/28]
22. Bohren MA, Vogel JP, Hunter EC, et al. The Mistreatment of Women during Childbirth in Health Facilities Globally: A Mixed-Methods Systematic Review. *PLoS Med* 2015;12(6):e1001847; discussion e47. doi: 10.1371/journal.pmed.1001847
23. National Academies of Sciences Engineering and Medicine. Crossing

the global quality chasm: Improving health care worldwide. Washington, DC, USA: National Academies of Sciences, Engineering, and Medicine, 2018.
24. Buckley R. World Development Report 2004: Making services work for poor people-Overview. Washington, DC, USA: The World Bank, 2003. http://documents.worldbank.org/curated/en/527371468166770790/, 2004.
25. Vogel JP, Bohren MA, Tuncalp, et al. Promoting respect and preventing mistreatment during childbirth. *BJOG* 2016;123(5):671-4. doi: 10.1111/1471-0528.13750 [published Online First: 2015/12/03]
26. Miller S, Lalonde A. The global epidemic of abuse and disrespect during childbirth: History, evidence, interventions, and FIGO's mother-baby friendly birthing facilities initiative. *Int J Gynaecol Obstet* 2015;131 Suppl 1:S49-52. doi: 10.1016/j.ijgo.2015.02.005
27. Van Belle S, Mayhew SH. Public accountability needs to be enforced -a case study of the governance arrangements and accountability practices in a rural health district in Ghana. *BMC Health Serv Res* 2016;16(1):568. doi: 10.1186/s12913-016-1836-1 [published Online First: 2016/10/13]
28. Freedman LP, Ramsey K, Abuya T, et al. Defining disrespect and abuse of women in childbirth: a research, policy and rights agenda. *Bull World Health Organ* 2014;92(12):915-7. doi: 10.2471/BLT.14.137869
29. White Ribbon Alliance. What Women Want: Demands for quality healthcare for women and girls. https://www.whiteribbonalliance.org/whatwomenwant/, Accessed November 2018.
30. World Health Organization and the United Nations Children's Fund (UNICEF). Declaration of Astana: From Alma-Ata towards universal health coverage and the Sustainable Development Goals. Astana, Kazakhstan: World Health Organization, 2018.

For peer review only

List of tables

Table 1. Facility managers' report on the availability of guidelines for identifying, responding and preventing abuses and community involvement in ensuring quality of care

Guideline or policy parameter in health facilities	Number of responses (%)		
	Bangladesh N=15	Ghana N=16	Tanzania N=12
A process for identifying abuses of client by provider	11 (73.3%)	13 (81.3%)	6 (50.0%)
"Whistle-blower" policy for peer reporting under cover	Not recoded	15 (93.8%)	3 (25.0%)
Women are encouraged to report provider abuses	9 (60.0%)	16 (100.0%)	4 (33.3%)
Response to most recent abuse case (number of cases)	1 case	5 cases	1 case
- *Investigated and penal actions taken internally*	0 (0.0%)	4 (80.0%)	1 (100.0%)
- *Investigated within facility but referred for action*	0 (0.0%)	1 (20.0%)	0 (0.0%)
- *Referred to external bodies for investigation & sanction*	1 (100.0%)	0 (0.0%)	0 (0.0%)
Measures taken to prevent future abuses[¥]			
- *Counselling and verbal warning*	1 (100.0%)*	5 (100.0%)	1 (100.0%)
Involves community stakeholders in quality improvement efforts	8 (53.3%)	12 (75.0%)	6 (50.0%)
Women's support groups exist in the catchment population	5 (33.3%)	10 (62.5%)	2 (16.7%)

**In Bangladesh, they raise awareness and supervise the staff who perpetrated the act.* [¥]*The assessment checked for other penal measures such as demotion of staff, reassignment/transfer, embargo on salary and interdiction but there was not reported in any of the facilities.*

For peer review only

Table 2. Care providers' report on availability of guidelines for respectful maternal and newborn care

Subject area	Providers' account on modalities for ensuring respectful maternity and newborn care	Number of responses (%)		
		Bangladesh N=158	Ghana N=86	Tanzania N=81
Policies on women's rights	Policy and procedure for addressing patient concerns	54 (41.1%)	61 (71.0%)	54 (66.7%)
	Policies on the rights of patients	83 (52.5%)	70 (81.6%)	57 (70.4%)
Staff training on Respectful care	Staff trained on how to treat childbearing women with respect	64 (40.5%)	60 (70.1%)	61 (75.0%)
Staff rating of RMC provided in their facility	Staff who rated their facility above 3 on a scale of 1 to 5 (5 being best) for care with respect and dignity they provide to women.	151 (95.6%)	86 (100.0%)	77 (95.1%)
Identification and response to Abuse	Process exist for identifying and reporting abuses during maternity and newborn care	46 (29.1%)	58 (67.8%)	41 (50.0%)
	Have encountered abuse victims in their maternity and newborn care practice	42 (26.6%)	28 (32.2%)	16 (19.8%)
	Staff who knew of 2 or more measures being implemented in their facilities to prevent abuses	31 (19.6%)	54 (62.7%)	57 (70.4%)
	Staff who mentioned 2 or more measures being implemented in their facilities to treat or rehabilitate victims of abuses	16 (10.1%)	17 (19.5%)	56 (69.0%)
	Community stakeholders' involvement in addressing disrespect and abuse of women during childbirth	49 (31.0%)	49 (57.5%)	27 (33.3%)
Payment for services	Women are expected to pay for normal deliveries	32 (20.3%)	7 (8.1%)	42 (51.9%)
	Women are expected to buy supplies for normal deliveries	132 (83.5%)	73 (85.1%)	61 (75.0%)
	Women are expected to pay before treatment in case of obstetric emergencies	19 (12.0%)	9 (10.3%)	27 (33.3%)

For peer review only

Table 3. Women's report of abuses, provider attitudes and overall satisfaction with care provided

Aspect of care	Modality for which women's perspective around facility experiences was assessed	Number of responses (%)		
		Bangladesh N=295	Ghana N=381	Tanzania N=173
Abuses during care-giving	Physical abuse from providers at the facility	6 (2.0%)	9 (2.4%)	2 (1.2%)
	Verbal abuse from providers at the facility	19 (6.4%)	25 (6.6%)	8 (4.6%)
	Sexual abuse from providers at the facility	2 (0.6%)	2 (0.7%)	1 (0.6%)
Provider relationship with caregivers	Their concerns were considered by providers in caring for them	224 (75.9%)	352 (92.4%)	108 (62.4%)
	Providers were responsive when asked for support	262 (88.8%)	366 (96.1%)	161 (93.1%)
	They were treated with respect by providers	266 (90.2%)	367 (96.1%)	168 (97.1%)
Satisfaction with care	Satisfied with attitude of providers	271 (91.7%)	353 (92.7%)	161 (93.1%)
	Satisfied with information received from providers on breastfeeding	191 (64.8%)	338 (88.7%)	132 (76.3%)
	Satisfied with information received from providers on family planning	38 (12.9%)	208 (54.6%)	77 (44.5%)

Table 4. Assessor observations of the provider attitudes during maternity care for women

Aspect of care	Modalities of care that were observed by assessors	Number of responses (%)		
		Bangladesh N=387	Ghana N=134	Tanzania N=120
Client requests	Women who made request for anything	14 (3.7%)	4 (3.0%)	4 (3.3%)
	- Those whose requests were refused with respect	1 (6.7%)	4 (100.0%)	3 (75.0%)
	Women who requested for a birth companion	286 (73.9%)	0 (0.0%)	2 (1.7%)
Payments at facility and effect on care	Women/families paid monies for some services	164 (42.4%)	19 (14.2%)	61 (50.8%)
	Women/families who complained about the payment	45 (11.6%)	1 (0.8%)	22 (18.3%)
	Women for whom services were withheld due to inability to pay	2 (0.5%)	0 (0.0%)	13 (10.8%)

For peer review only

Legend of figures

Figure 1. Availability of client toilets with soap and water for handwashing and its cleanliness

Figure 2. Women's reported experience of care at maternity units

Figure 3a-3d. Assessors' observations on women's maternity care experiences during provider interactions in health facilities

Figure 4. Mothers' satisfaction with care received in the health facilities during childbirth in the 3 countries

For peer review only

For peer review only

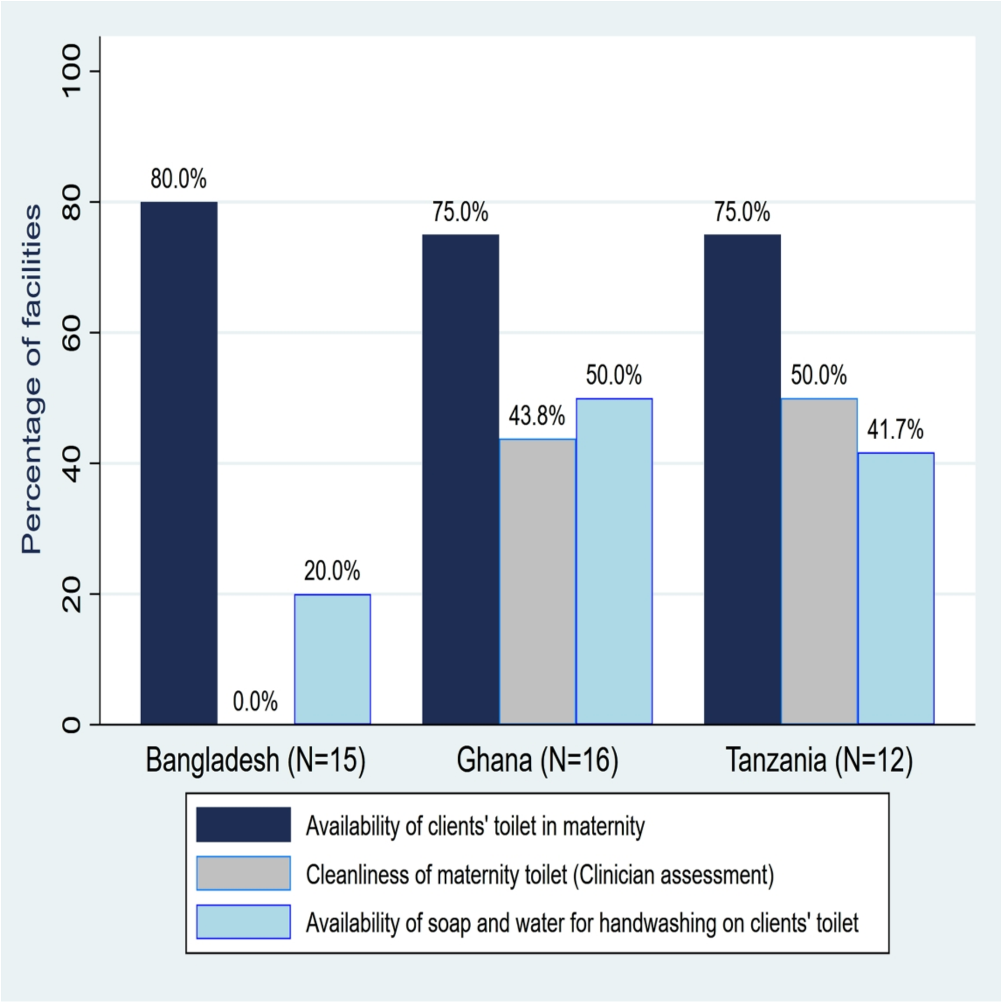

Figure 1. Availability of client toilets with soap and water for handwashing and its cleanliness

90x90mm (600 x 600 DPI)

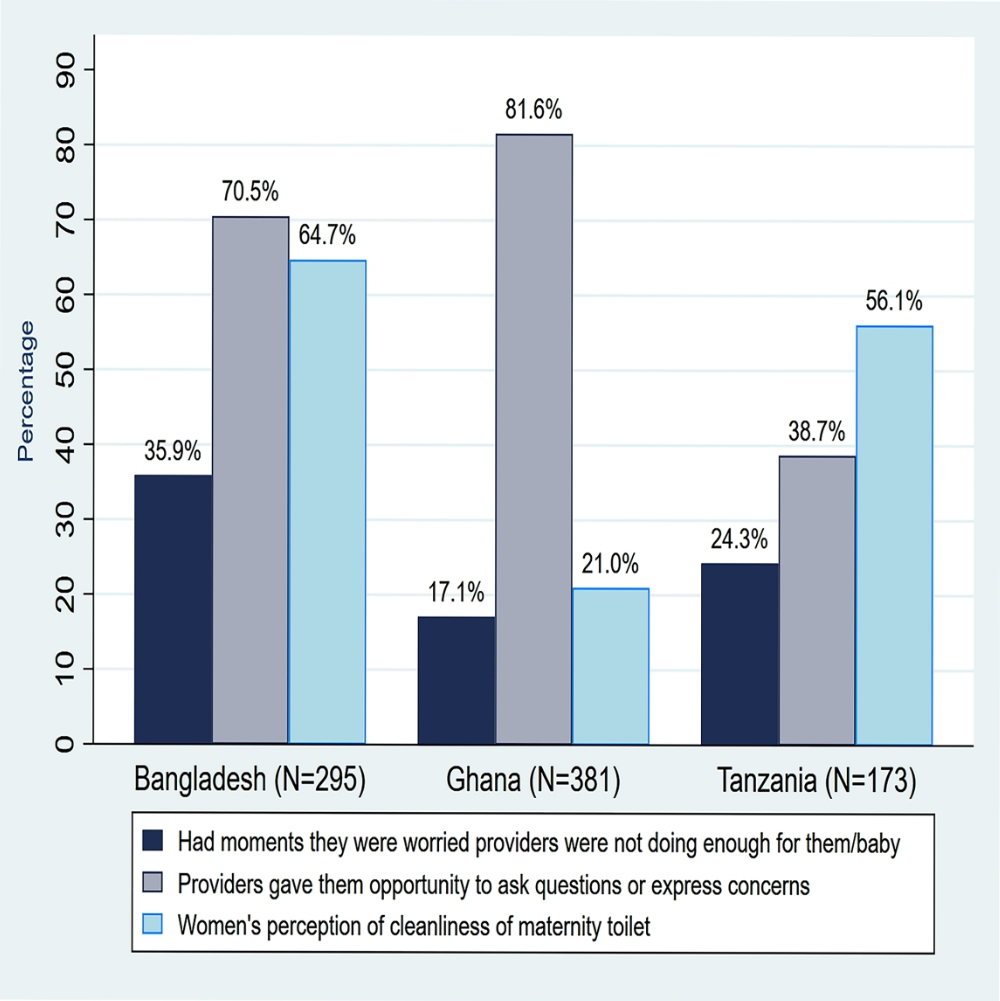

Figure 2. Women's reported experience of care at maternity units

90x90mm (600 x 600 DPI)

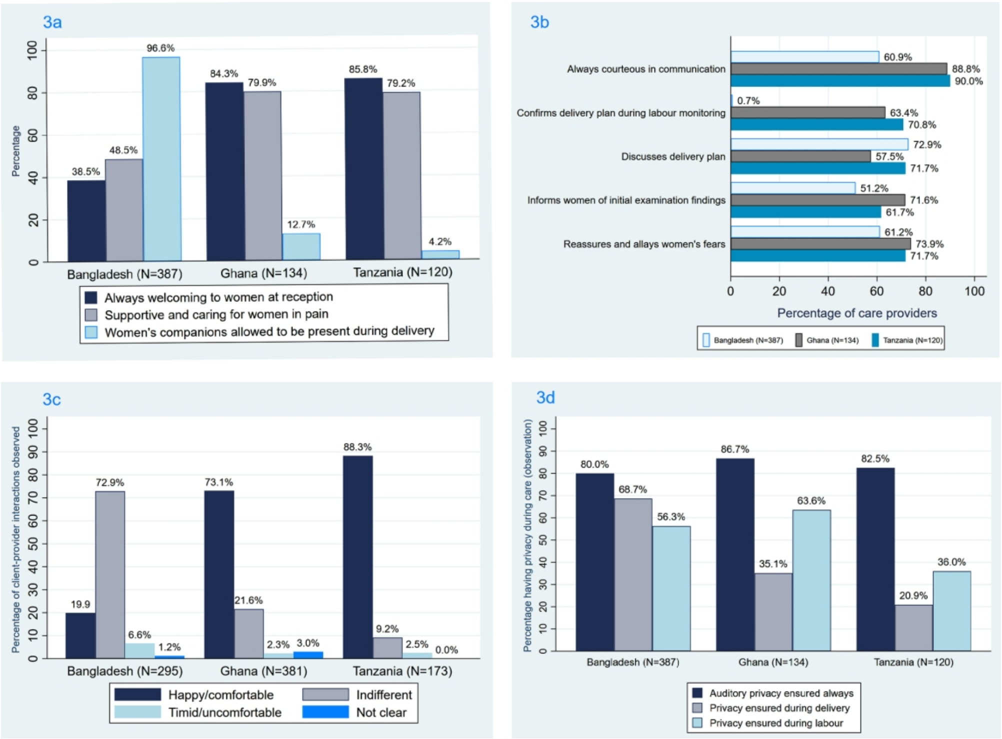

Figure 3a-3d. Assessors' observations on women's maternity care experiences during provider interactions in health facilities

90x66mm (600 x 600 DPI)

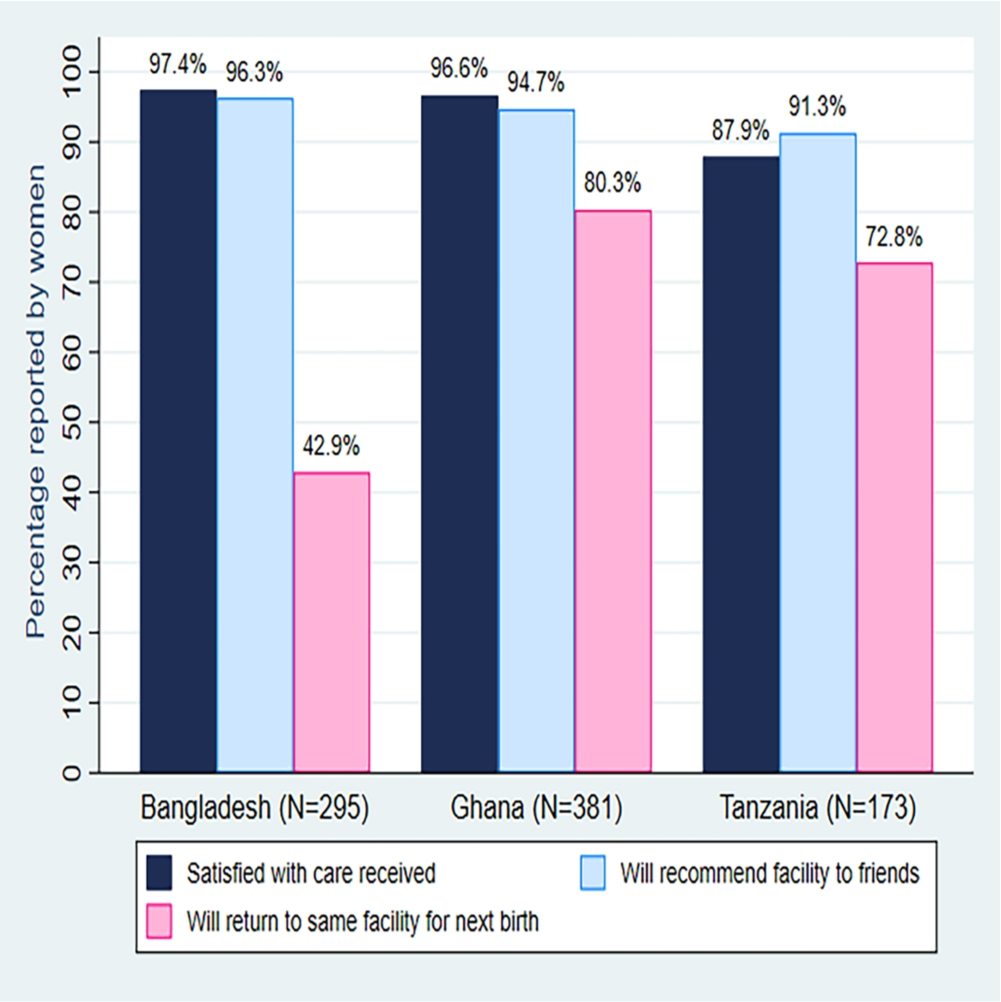

Figure 4. Mothers' satisfaction with care received in the health facilities during childbirth in the 3 countries

90x90mm (600 x 600 DPI)

STROBE guideline checklist

**STROBE guidelines checklist**	**Page number**
Title	1
Abstract	2
**Introduction**	**4**
Objectives	4
**Methods**	
Study design	4
Setting	5
Participants	5
Variables	6
Data sources / measurement	6
Bias	6
Study size	6
Quantitative variables	6
Statistical methods	7
**Results**	
Participants	8
Descriptive data	8
Outcome data	8-11
Main results	8-11
Other analysis	Not applicable
**Discussion**	
Key results	11
Limitations	12
Interpretation	11-12
Generalizability	12
**Other information**	
Funding	13

For peer review only

BMJ Open

Respectful Maternity Care delivered within health facilities in Bangladesh, Ghana and Tanzania - A cross-sectional assessment preceding a quality improvement intervention

Journal:	*BMJ Open*
Manuscript ID	bmjopen-2020-039616.R2
Article Type:	Original research
Date Submitted by the Author:	30-Dec-2020

Complete List of Authors:	Manu, Alexander; University of Ghana College of Health Sciences, Epidemiology and Disease Control/School of Public Health; Liverpool School of Tropical Medicine, Centre for Maternal and Newborn Health<br>Zaka, Nabila; UNICEF<br>Bianchessi, Christina; UNICEF,<br>Maswanya, Edward; NIMR<br>Williams, John; Navrongo Health Research Centre<br>Arifeen, Shams E.; ICDDR B, Maternal and Child Health Division (MCHD)

**Primary Subject Heading**:	Global health
Secondary Subject Heading:	Epidemiology, Health policy, Health services research, Patient-centred medicine
Keywords:	Quality in health care < HEALTH SERVICES ADMINISTRATION & MANAGEMENT, PUBLIC HEALTH, Maternal medicine < OBSTETRICS, Health policy < HEALTH SERVICES ADMINISTRATION & MANAGEMENT

SCHOLARONE™
Manuscripts

I, the Submitting Author has the right to grant and does grant on behalf of all authors of the Work (as defined in the below author licence), an exclusive licence and/or a non-exclusive licence for contributions from authors who are: i) UK Crown employees; ii) where BMJ has agreed a CC-BY licence shall apply, and/or iii) in accordance with the terms applicable for US Federal Government officers or employees acting as part of their official duties; on a worldwide, perpetual, irrevocable, royalty-free basis to BMJ Publishing Group Ltd ("BMJ") its licensees and where the relevant Journal is co-owned by BMJ to the co-owners of the Journal, to publish the Work in this journal and any other BMJ products and to exploit all rights, as set out in our licence.

The Submitting Author accepts and understands that any supply made under these terms is made by BMJ to the Submitting Author unless you are acting as an employee on behalf of your employer or a postgraduate student of an affiliated institution which is paying any applicable article publishing charge ("APC") for Open Access articles. Where the Submitting Author wishes to make the Work available on an Open Access basis (and intends to pay the relevant APC), the terms of reuse of such Open Access shall be governed by a Creative Commons licence – details of these licences and which Creative Commons licence will apply to this Work are set out in our licence referred to above.

Other than as permitted in any relevant BMJ Author's Self Archiving Policies, I confirm this Work has not been accepted for publication elsewhere, is not being considered for publication elsewhere and does not duplicate material already published. I confirm all authors consent to publication of this Work and authorise the granting of this licence.

For peer review only

Respectful Maternity Care delivered within health facilities in Bangladesh, Ghana and Tanzania - A cross-sectional assessment preceding a quality improvement intervention

Alexander Manu[1,2], Nabila Zaka[3], Cristina Bianchessi[3], Edward Maswanya[4], John Williams[5] and Shams El Arifeen[6]

[1] School of Public Health, University of Ghana, Legon, Accra
[2] Centre for Maternal and Newborn Health, Liverpool School of Tropical Medicine, Liverpool, United Kingdom
[3]United Nations Children's Fund/HQ, New York, United States of America
[4]National Institute for Medical Research, Dar es Salaam, Tanzania
[5]Navrongo Health Research Centre, Navrongo, Ghana
[6]International Centre for Diarrhoeal Diseases Research, Bangladesh, Dhaka, Bangladesh

Address of authors:

AM – makmanu128@gmail.com
NZ – nzaka@unicef.org
CB - c.bianchessi@gmail.com
EM - edward19631@hotmail.com
JW - John.Williams@navrongo-hrc.org
SA - Shams@icddrb.org

‡Correspondence to:
Dr Alexander Manu
Department of Epidemiology and Disease Control of the School of Public Health
University of Ghana
Legon, Accra, Ghana
Email: makmanu128@gmail.com

Keywords: Respectful care, Dignified care, Quality, Maternal. Newborn, Health facility.

Word Count: 5100

For peer review only

ABSTRACT

Objective: To assess respectful maternity care (RMC) in health facilities.

Design:Cross-sectional study

Setting: Forty-three(43) facilities across 15 districts in Bangladesh(15), Ghana(16) and Tanzania(12).

Participants:Facility Managers; 325 providers (nurses/midwives/doctors)-Bangladesh(158), Ghana(86) and Tanzania(81) and 849 recently-delivered women-Bangladesh(295), Ghana(381) and Tanzania(173)-were interviewed. Observation of 641 client-provider interactions was conducted–Bangladesh(387), Ghana(134) and Tanzania(120).

Assessment:Trained social-scientists and clinicians assessed infrastructure, policies, provision and women's experiences of RMC(emotional support, respectful care and communication).

Primary outcome:RMC provided and/or experienced by women.

Results:Three(20%) facilities in Bangladesh, four(25%) in Ghana and three(25%) in Tanzania had no maternity clients' toilets and one-half had no handwashing facilities. Policies for RMC such as identification of client abuses were available: 81%(Ghana), 73%(Bangladesh) and 50%(Tanzania) but response was poor. Ninety-four(60%) Bangladeshi, 26(30%) Ghanaian and 20(25%) Tanzanian providers were not RMC-trained. They provided emotional support during labour care to 107(80%) women in Ghana, 95(79%) in Tanzania and 188(48.5%) in Bangladesh and were often courteous with them-236(61%) in Bangladesh, 119(89%) in Ghana and 108(90%) in Tanzania. Due to structural challenges 169(44%) women in Bangladesh, 49(36%) in Ghana and 77(64%) in Tanzania had no privacy during labour. Care was refused to 13(11%) Tanzanian and two Bangladeshi women who could not pay illegal charges. Twenty-five(7%) women in Ghana, nine(6%) in Bangladesh and eight(5%) in Tanzania were verbally abused during care. Providers in all countries highly-rated their care provision (95%-100%) and 287(97%) of Bangladeshi women, 368(97%) Ghanaians and 152(88%) Tanzanians reported "satisfaction" with the care they received. However, based on their facility experiences, significant($p<0.001$) percentages-20%(Ghana) to 57%(Bangladesh)-will not return to the same facilities for future childbirth.

Conclusions:Facilities in Bangladesh, Ghana and Tanzania have foundational systems that facilitate RMC. Structural inadequacies and policy gaps pose challenges. Many women were, however, unwilling to return to the same facilities for future deliveries although they (and providers) highly rated these facilities.

For peer review only

Article Summary

Strengths and limitations of this study

- This study is from one the largest quality improvement interventions in LMICs settings as it harmonised the methods and developed common tools/instruments to assess respectful maternity care in 43 health facilities across the three countries.
- The study employed a variety of approaches including direct observations, interviews and records review, and triangulated the findings to comprehensively assess respectful maternity care provided at the health facilities both in the past and at present.
- The cross-sectional design and the short duration of contact with facilities only allowed for limited aspects of respectful care to be assessed but was a pragmatic consideration in order not to unduly interrupt care provision.
- Women's reported experiences and providers' reported practices may introduce biases; observing provider-client interactions may likely find improved quality of care than what is routinely provided to clientele (Hawthorne effect) and therefore, inadequacies in quality that were identified during the observations may under-represent the true extent of sub-optimal provision of respectful care.
- The findings may have limited external validity due to UNICEF's equity focus in selecting areas of work in the respective countries; most, but not all, of the assessed facilities were in the most under-served districts and this may represent a selection bias in the context of the country.

For peer review only

INTRODUCTION

In 2015, World Health Organization (WHO) issued a statement calling for global actions to prevent and eliminate disrespect and abuse during facility-based childbirth and emphasized women's right to dignified and respectful care throughout pregnancy and childbirth[1]. Facility birth rates have increased due to increased awareness, demand and access to maternity services[2]. Approximately 6.5 million more women delivered with skilled providers in 2015 and, across 60 Countdown to 2030 countries, the median skilled attendance rate increased from 65% in 2015 to 77% in 2017[3]. However, optimism around the increasing trend in facility deliveries must be cautious because deaths among pregnant women and their babies (born and unborn) have declined at a much slower pace, confirming that, unless coupled with care of the requisite quality, outcomes for women and their babies may be poor[4-8] and communities may lose trust in facilities[9 10].

D'Oliveira et al[11] and other authors found that pregnant women were abused within health facilities during childbirth; the care they receive is not respectful or dignified[11-13]and does not place any value on their expectations. The WHO framework of 2016 therefore defined eight standards for improving the quality of maternal and newborn health (MNH) care in health facilities along two domains – provision and experience of care[14]. It recognises the need to amplify the voices of women from low- and middle-income settings (LMICs) in the design, content and delivery of the care they receive around childbirth to ensure that it respects their dignity, preferences and aspirations[15]. The eight WHO standards were not systematically tested in any LMIC. Moreover, it is not fully described how respect and dignity of care can be objectively assessed. Tools such as the Mothers on Respect index have not been tested in LMICs[16].

As part of a Mother and Baby Friendly Hospital Initiative (MBFHI), UNICEF and partners in the Ministries of Health of Bangladesh, Ghana and Tanzania are working within selected districts to effectively improve breastfeeding and MNH outcomes. The partnership used this platform to implement a quasi-experimental, pre-post study to test the feasibility of implementing the WHO standards within these LMICs and to answer these critical questions: how pervasive is disrespectful and abusive care within health facilities in LMICs? Do facilities have and enforce conducive policy environments and/or guidelines that enable provision and experience of respectful maternity care (RMC) and what are (and can we rely on) women's perspectives on the dignity around the care they experience?

Our objective was to assess RMC from provider perspectives and as experienced by clients in labour wards within the three countries. We also aimed to assess the implications of these on women's satisfaction with the care. The manuscript therefore presents findings from a baseline assessment describing existing provisions, health worker delivery and families' perceptions of RMC prior to the implementation of the standards. The results will contribute to the continuing global discussions around how to effectively assess respect and dignity in the provision and experience of maternity care.

METHODS

Study Design and Setting

This is a cross-sectional study, nested within the quasi-experimental pre-post evaluation of the Every Mother Every Newborn Quality Improvement (EMEN-QI) intervention. The protocol for the evaluation

For peer review only

of the EMEN-QI intervention has been published[17]. This study had four components: inventory to check the availability of clean toilet and handwashing facilities, policies, protocols and guidelines for providing RMC; questionnaire and qualitative interviews with facility staff and maternity clients; structured observation of maternity care provided to clients and review of client medical records for documentation of RMC provided in the past.

It was carried out by three research institutions from the respective countries–International Center for Diarrhoeal Diseases Research in Bangladesh (ICDDR,B), Navrongo Health Research Centre (NHRC), Ghana Health Service and the National Institute for Medical Research (NIMR), Tanzania. It was conducted between May and August 2016 in 43 health facilities: 15 in Bangladesh, 16 in Ghana and 12 in Tanzania, comprising 1 regional and 12 district hospitals and 30 health centres (HCs)/upazilla health complexes (UHCs). Teams of 2-4 clinician research assistants (CRAs) and research social scientists/anthropologists (RSAs) carried out the assessment in each facility. These teams were trained by the principal investigators in each country under the direct supervision of the lead author. The training was followed by pretesting exercises which included hospital visits until there was consensus on the observations made for client-provider interactions.

In Bangladesh, three hospitals and twelve UHCs in Kurigram, Lalmonirhat and Gaibandha districts of the Rangpur division were involved. In Ghana, the regional hospital of the Upper East Region (UER), five district hospitals and ten health centres in eight districts (Bawku, Kassena-Nankana East and Bolgatanga Municipalities and Bawku West, Bongo, Kassena-Nankana West and Builsa North Districts) were covered. For Tanzania, the assessments were done in four hospitals and eight health centres within Njombe Town Council, Makete, Ludewa and Wanging'ombe districts of the Njombe region. These facilities provide basic/comprehensive emergency obstetric care (BEmOC/CEmOC) services and were selected in consultation with the ministries of health of the respective countries to reflect high average caseload, relatively poor maternal and newborn health indices and nationally-representative human resource availability.[17] All district hospitals, UHCs in Bangladesh and one HC Tanzania provided CEmOC services.

Participants

To cover provision and experience of care, respondents for the study included facility managers and "in-charges", providers who directly cared for women in labour for the two weeks of the assessment, recently-delivered women and their birth companions. The health records of all women, who were seen and cared for at the maternity in the 3-6 months prior to the assessment, were reviewed and data abstracted.

Informed Consent

We consented all participants to participate in the study. Research Assistants read out or gave an information sheet to all participants explaining the purpose of the assessment in a language of their choice. Each participant was allowed to ask for clarifications on the study. They were informed that there will be no direct benefits for participation but the information they provide will help improve the quality of maternity care provided in facilities. They were assured of their right to withdraw participation at any time without affecting their position in the facility or care they received and that information provided will be treated as confidential. Agreement to participate was indicated with a

For peer review only

signature/thumbprint. No respondent refused participation in the study. All interviews lasted 45-60 minutes.

Ethics Approval

The study protocol was approved by institutional ethics review boards (IRBs) in all three countries: ICDDR,B in Bangladesh (PR-16024, 1st June 2016), Ghana Health Service (GHS) in Ghana (NHRCIRB226, 6th April 2016) and NIMR in Tanzania (NIMR/HQ/R.8a/Vol. IX/2176, 11th April 2016). Clearance was sought from the facility managers for the study in the respective facilities.

Patient and Public Involvement

The study tools and instruments were pretested in all the three countries to solicit facility-user input into the strategy for data collection. The data collection involved patients or maternity clients who have been discharged from the facilities as well as their companions who accompanied them to the facility for the delivery. Their identities were protected, and their confidentiality and privacy were ensured for all the information they provided. Their contributions have been cited in the acknowledgements.

Assessment of Respectful Maternity Care (RMC)

RMC was assessed along the dimensions of the three components in the WHO framework - emotional support, respectful communication and respectful and dignified care - from the perspectives of the provider, the women (clients) and independent informed-assessors (CRAs). These were supplemented with data on the availability of the right policy environment and facility provisions (e.g. handwashing) for RMC.

After facility entry, CRAs interviewed facility superintendents/"in-charges" and assessed whether the facility had specific protocols and guidelines for ensuring RMC including training schedules for staff at maternity units. Using a semi-structured questionnaire, the CRAs assessed whether facilities had systems for identifying abuses of pregnant women seeking or receiving care including "whistle blower" policies where care providers could report client abuses perpetrated by their peers under protected or concealed identities. Respondents were asked how they involved communities in defining and providing quality and woman-centred maternity care. We considered the availability of clean client toilets on the maternity as a basic necessity in providing RMC and so CRAs checked availability of maternity ward toilets and rated their cleanliness based on agreed criteria.

CRAs also interviewed care providers in the maternity wards on any formal RMC training they received, any encounter with situations of client abuse in facilities and management responses to abuses. Care providers included specialists, doctors, midwives and staff nurses. Selection was biased towards staff who play key leadership or decision-making roles in facilities (e.g., matrons who provide care and enforced guidelines and regulations) or served for more than six months.

The CRAs also passively observed client-provider interactions as women navigated through all the points of care provision within facilities–from reception through till discharge after delivery. These provided first-hand experiences of auditory and visual privacy and provider-client communication such as explanation of the care plan and the involvement of clients in their own care. They checked whether providers ensured a conducive atmosphere for women and their companions to ask

For peer review only

questions on the care they received; support (emotional and other) for women during childbirth; and any abuses. They reviewed women's records to assess documentation of care.

RSAs identified isolated, comfortable and conducive spaces within facility to interview recently-delivered women, who have been discharged from facilities, on their experiences of the maternity care provided. All women who have been in the facility for at least six hours and consent to participate were interviewed irrespective of birth outcomes. This was because women who present in labour were observed for at least 6 hours after the birth before discharge, creating opportunity for them to interact with providers. The RSAs ensured that respondents were not seen or heard during the interview. The interview covered women's perspectives on provider communications, responsiveness, respect for their choices and preferences, visual and auditory privacy during care provision, demand for informal or formal payments and withholding of care if such demands were refused, any physical, sexual or verbal abuses during women's stay in the facility. Clients' impressions and satisfaction with the maternity care they experienced were also elicited but aside that, they were asked whether their experiences will make them recommend the facility to relatives or friends for maternity care or whether they themselves will like to return to the same facility for their next childbirth.

RSAs also interviewed any persons accompanying the women during their stay in the facility for their independent perspectives on the care provided to their relative or friend. RSAs asked whether providers demanded or collected payments for services or whether any service was withheld for any reason including inability to pay.

Quality assurance and data processing

Stringent uniform quality assurance (QA) processes were implemented starting from the field through to the data processing units of the research institutions. Team coordinators liaised between fieldworkers, facility leadership and data centres. Data collection was paper-based in Ghana and Bangladesh but electronic in Tanzania. At the end of each day, research teams met and peer-reviewed each other's forms for completeness. Team coordinators re-checked all forms to ensure blanks or inconsistencies were identified and resolved. All checked forms were batched, logged into batch control sheets and transmitted to computer centres for processing.

In Ghana and Bangladesh, two data entry clerks independently entered the data and the two data streams were compared for agreement. Any disagreements were resolved with the source questionnaire by data managers who then run range and consistency and data integrity checks to generate exception reports for the study investigators to review and resolve. In Tanzania, data were directly transmitted to central servers for review by data managers. Where required, forms with challenges were sent back to the field for resolution. Cleaned data were securely stored on password-protected servers. A copy of the final data was shared with UNICEF/HQ.

Data analyses

Data were transferred to Stata version 14.1 for analyses. Data were represented with tabular, numerical and graphical methods. Proportions, means(sd) and medians (IQR) were estimated for the outcome analyses. Z-tests were performed to assess differences in proportions and χ^2 tests for association between variables. All tests were significant at the 5% level and 95% confidence intervals were constructed around point estimates where necessary. Responses to open-ended questions were

For peer review only - http://bmjopen.bmj.com/site/about/guidelines.xhtml

coded into themes in NVIVO 12.0 after repeated readings using the framework approach. Qualitative analyses involved exploration of relationships between themes and were triangulated to the close-ended questions to facilitate their contextual interpretations.

Coordination

UNICEF coordinated the uniform assessment across countries. A consultant (AM) facilitated a UNICEF-organised design meeting and training in New York with principal investigators (PIs). Skype conference calls were organized every week to agree harmonised timelines for implementation and update on progress. The coordinated approach allowed for common solutions to challenges encountered and for the consultant to visit each country during the training and initiation of data collection.

RESULTS

Health facility managers in all 43 facilities across the three countries were interviewed. There were 325 care provider interviews with nurses, midwives and doctors comprising 158 in Bangladesh, 86 in Ghana and 81 in Tanzania. Questionnaires were administered by social scientists/anthropologists to collect data from a total of 849 recently-delivered women: 295 from Bangladesh, 381 from Ghana and 173 from Tanzania. Observation of 641 client-provider interactions was conducted by CRAs – 387 in Bangladesh, 134 in Ghana and 120 in Tanzania.

Availability of dedicated clean maternity toilet as part of respectful maternity care

Clean toilets with handwashing facilities were inadequate in the maternity units in all three countries (figure 1). Three (20%) facilities in Bangladesh, four (25%) in Ghana and three (25%) in Tanzania did not have client toilets in the maternity. Where they existed, they were judged as unclean by study clinicians-6(50%) in Tanzania, 9(56%) in Ghana and 15(100%) in Bangladesh-and without soap and water for handwashing-7 (58%) in Tanzania, 8 (50%) in Ghana and 12 (80%) in Bangladesh. Women's perceptions on the cleanliness of the toilets contrasted with these clinician observations. Figure 2 shows that only 104(35%) women interviewed in Bangladesh and 301 (79%) in Ghana thought the toilets were unclean.

Availability of the right policy environment for respectful maternity care

Majority (13 representing 81.3%) of facility managers in Ghana, compared to 11 (73.3%) in Bangladesh and 6 (50.0%) in Tanzania, reported that clear processes existed for identifying client abuses (Table 1). In Ghana, all but one facility had a "whistle-blower" policy for reporting abuses, and all facilities (100%) actively encourage women to report any provider abuses. In Tanzania only one in four facilities had a "whistle-blower" policy and one in three encourage women to report abuses. Data on whistle blower policies were not captured in Bangladesh although, in qualitative interviews, staff suggested that mechanisms for making anonymous complaints using mobile SMS existed, were captured onto the district health information systems (DHIS-2) platform and followed-up regularly.

Consistent with facility managers' reports, assessors found that, of all three countries, most health facilities in Ghana had policies on women's rights during care at the facility. Sixty-one (71%) providers in Ghanaian facilities reported that they had policies on addressing client concerns and 70 (82%) reported that policies existed on the rights of clients who sought maternity care (table 2). In Bangladesh, only 54(41%) of providers interviewed reported the existence of policies to address client

For peer review only

concerns and 83(53%) reported policies existed on women's rights, although the staff could not show copies of the policy documents to the assessors during the visits. In Tanzania, 54(67%) providers reported that their facilities had policies to address client concerns whilst 57(70%) reported that policies existed on women's rights whilst receiving care (table 2)

Training of providers in respectful maternity care

Specific training on how to provide maternity care with dignity and respect was not provided to 30%-60% of providers across the three countries. In Bangladesh, 127(80%) providers could not mention two measures being implemented in their facilities to prevent abuses of women during maternity care (Table 2). There were 32(37%) providers in Ghana and 24(30%) in Tanzania who could also not mention two or more measures. A substantial proportion-69(80%) in Ghana and 142(90%) in Bangladesh-did not know of two or more measures in place to treat or rehabilitate victims of abuse. Meanwhile, table 2 also shows that almost all of these providers highly rated the respect and dignity in the care they provided, scoring it above 3 on a scale of 1 to 5, 5 being the best-151 (96%) of them in Bangladesh, 86 (100%) in Ghana and 77 (95%) in Tanzania.

Women's reported experience of maternity care and how it compares with assessors' observations

Whilst most women reported that they were happy with the attitude of providers (table 3), they also said providers were not doing enough for them and/or their babies and were not given the opportunity to express these concerns (figure 2). For example, in Tanzania, although 168 (97.1%) of women reported that providers treated them with "respect" (table 3) and 161 (93.1%) said providers were responsive to them when they asked for support, figure 2 also shows that about a quarter of them 42 (24.3%) reported about moments when they were worried that the providers were not doing enough for them. Moreover, 106 (61.3%) reported that they had no opportunity to express these concerns or ask questions. The trends were similar in Ghana and Bangladesh; in Bangladesh, for instance, 266 (90.2%) women reported being treated with "respect" but 106 (35.9%) had moments they were concerned that the providers were not doing enough for them and 87 (29.5%) thought they were not given the opportunity to express these concerns.

During the observation of client-provider interactions by the study assessors varying trends were found between the three countries. Figures 3a and 3b show that, in Ghana and Tanzania, at reception, the providers were mostly welcoming-113 (84%) and 103 (86%) respectively-and were supportive and caring when women were in labour pain 107 (80%) and 95 (79%) respectively. The assessors also found, on a Likert scale between "never", "only sometimes", "mostly" to "always", providers were "always courteous" in communication with the women-119 (89%) in Ghana and 108 (90%) in Tanzania). In the two countries, providers informed 62%-72% of women about their initial examination findings, discussed delivery plans with 52%-72% of them, reassured them and allayed their fears in over 70% of cases and confirmed delivery plans during labour monitoring in 63%-71% of cases. However, figure 3c shows that, in these same countries, 12%-27% of respondents were not "comfortable" talking to the providers and 2.3%-2.5% were visibly uncomfortable or looked timid. Only 3% of women in the two countries made specific requests from the providers and none in Ghana could request for a birth companion (Table 4).

In contrast, the findings were different in Bangladesh as uptake of these modalities in RMC were lower. Figures 3a and 3b show that providers were "always welcoming" to 149 (36%) of women, were

For peer review only

supportive and caring during labour pains in 188 (49%) and just about half 198 (51%) were told examination findings at initial exams. Though the delivery plan was discussed with 282 (73%) of the women, during labour monitoring, these plans were confirmed with only 3(0.7%) of them. Table 4 shows that about 14 (4%) of women in Bangladesh made specific request from the providers and though all were refused, it was only in a single case that the refusal was done respectfully. Figure 3c shows that 25 (7%) of women in Bangladesh felt uncomfortable and timid when talking to the providers, about three-times those in Ghana and Tanzania.

Companion at birth

No woman in Ghana and only two (2%) in Tanzania reported requesting a birth companion for the delivery (Table 4). This contrasts with the 286 (74%) in Bangladesh. However, in figure 3a, women's companions were allowed to be present for 17 (13%) of women in Ghana, 5 (4%) in Tanzania and 374 (97%) in Bangladesh.

Verbal, physical or sexual abuse of women during maternity care

Facility managers interviews (Table 1) showed that cases of abuse of women during maternity care were reported but measures to prevent recurrence were weak. Managers were aware of recent (less than 2 weeks) cases of abuse in their facilities. Five cases of abuse were reported in Ghana compared to only one each in Bangladesh and Tanzania. Staff also reported encounters with victims of abuse in their practice. About 16 (20%) providers in Tanzania, 42 (27%) in Bangladesh and 27 (32%) in Ghana had encountered victims of abuse in their practice but up to 70% of providers between the three countries did not personally know of any existing systematic processes for identifying and reporting abuses within their facilities although managers reported that these processes existed. Meanwhile, women reported that they were physically, verbally or sexually abused by providers during care (Table 3). The commonest form of abuse was verbal; on average, 6% of women in all three countries experienced this form of abuse (5% in Tanzania, 6% in Bangladesh and 7% in Ghana). Sexual abuses were the rarest with only 1 or 2 women reporting the experience in each country. On average, 2% of the women also experienced physical abuses from providers during their stay in the facility.

When these abuses occurred, facilities in Ghana and Tanzania conducted internal investigations and the only action taken to prevent future occurrences was counselling or verbal warnings to the staff involved (5 of the 6 cases). In comparison, the only recent case in Bangladesh was referred to an external body for investigation and action.

Privacy of care

During assessors' observations of client-provider interactions, of the three countries, auditory privacy was ensured in over 80% of interactions (Figure 3d). Privacy during labour was ensured mostly in Ghana where there was privacy in 64% of the 134 client-provider interactions observed. In Bangladesh, privacy in labour was ensured for 218 (56%) of interactions and it was for 43 (36%) in Tanzania. During delivery care privacy was least ensured in Tanzania (21% of 120 observations). It was 35% in Ghana and 69% in Bangladesh.

Demand for payment and withholding of delivery care services for inability to pay

For peer review only

Consistent with findings from CRA observations of client-provider interactions, providers in all three countries reported that women were made to pay monies for supplies and/or delivery, even in obstetric emergencies (table 2). Charging fees was most common within facilities in Tanzania where 42 (52%) providers reported that women were made to pay fees for normal deliveries. Twenty-seven (33%) providers in Tanzania said these payments were also expected for obstetric emergencies. The proportion of providers reporting these payments were relatively lower in Bangladesh where 32 (20%) reported payments for normal deliveries and 19 (12%) in emergencies. In Ghana, where a National Health Insurance Scheme covers the cost of facility births, 7 (8%) providers reported that some illegal fees were still charged for normal deliveries and 9 (10%) said there were fees charged for obstetric emergencies. Seventy-three (85%) providers in Ghana, 132 (83%) in Bangladesh to 61 (75%) in Tanzania reported that women had to buy supplies for normal deliveries in the facility.

During observation of client-provider interactions, study assessors found that providers demanded monies from women for maternity services. This practice was most common in Tanzania where the demands were made for 61 (51%) cases observed, followed by 164 (42%) in Bangladesh and 19 (14%) in Ghana. Maternity services were sometimes withheld from women when they were unable to pay and, only a few women complained about such payments. During observations, one mother in Ghana, 45 (12%) in Bangladesh and 22 (18%) in Tanzania were seen complaining about the charges. It is noteworthy that some delivery care services were withheld from women (11% in Tanzania and 0.5% in Bangladesh) because inability to pay. No such service was withheld in Ghana.

Community involvement providing respectful maternity care

Managers of facilities reported that six (50%) facilities in Tanzania, eight (53%) in Bangladesh twelve (75%) in Ghana involved community stakeholders in deliberations around ensuring RMC (table 2). Facility providers corroborated this. When care was abusive or not respectful, 49 (31%) providers in Bangladesh, 27 (33%) in Tanzania and 49 (58%) in Ghana reported that their facility involved community stakeholders in addressing it.

Overall satisfaction with the maternity care at health facilities

On a scale of 1 to 5 (5 being the best) in terms of RMC provided to women, all (100%)providers from Ghana, 151 (96%) from Bangladesh and 77 (95%) from Tanzania ranked their facilities 3 and above. Over 90% of women from all countries also reported satisfaction with the attitude of the providers - 271 (91.7%) in Bangladesh, 353 (92.7%) in Ghana and 161 (93.1%) in Tanzania (Table 3). Figure 4, however, shows that majority of women expressed satisfaction with the maternity care they received at facilities-287 (97%) in Bangladesh but, on the contrary, only 106 (43%) will to return to the same facilities for future maternity care and this difference was statistically significant ($p<0.001$). Similarly, 386 (97%) in Ghana expressed satisfaction with the care but only 305 (80%) will return to the same facility for the next delivery whilst in Tanzania, 152 (88%) were satisfied but and only 126 (73%) will return to the same facilities in the future for maternity care showing differences that were statistically significant ($p<0.0001$). Meanwhile these same women would recommend the facility to a relative or friend for maternity care even if they themselves will not do same in the future-96% in Bangladesh, 95% in Ghana and 91% in Tanzania.

DISCUSSION

For peer review only

Though respectful maternity care is a relatively new and developing concept in many LMICs, our assessment in the three countries found that the infrastructure, policies on caregiving and staff readiness for providing RMC were in place to build upon. Prior to implementing EMEN-QI, uptake of practices around the three RMC domains (effective communication, supportive care and dignified and respectful care) was variable across the three countries but generally high. Three-quarters and more of the facilities had separate toilets in the maternity for women and half of these had handwashing facilities. Providers' and managers' reports showed that approximately 70% facilities in Ghana and Tanzania had policies on patients' rights and procedures for addressing their concerns. Only in 40%-50% in Bangladesh had same and without this right policy environment, women in Bangladesh did not have good reception, examination findings were not communicated to them, their concerns were not considered in the care they received and they were not supported during labour. Structural inadequacies did not allow for privacy during maternity care for women and birth companions were also not present especially in Tanzania and Ghana. Moreover, illegal payments were demanded from women for delivery care and services were sometimes withheld due to inability to pay. With the right processes for ensuring patient rights, including "whistle blowers" policy as in Ghana, abuse reporting was higher. Facility providers over-rated RMC they provided as compared to what was observed. Between 50% to 75% of facilities already involve community stakeholders in addressing RMC experiences of women. Although most women across the three countries reported being "satisfied" with care provided at facilities, they will not come back to the same facilities for their next delivery based on their experiences.

Our findings align with those from earlier studies suggesting that assessing care satisfaction with questions that merely ask whether women or providers were satisfied with care received or provided was unreliable[9]. Our results show that whilst 88%-97% of women and providers highly rated their satisfaction with care received or provided, perhaps a probable indicator of satisfaction with care may be the significant 20% to 58% ($p<0.0001$) of women who reported that, based on the maternity care experienced, they will not return to the same facilities for birth in the future. This apparent inconsistency between women's reported satisfaction with care and desire to return to same facility has not often been assessed although alluded to when researchers find unbelievably high reported level of satisfaction under situations that did not appear to merit same[9 10]. Lack of respectful and dignified care are known barriers to subsequent careseeking[18]. This indicator will require testing in robust studies.

There appeared differences in observed provider attitudes between the South Asian (SA) and sub-Saharan African (SSA) facilities in that, apart from Bangladesh, Figure 3a shows that providers were mostly welcoming to women and supported them during labour pains. With only 39% of providers welcoming women and 48% supporting in Bangladesh compared to over 80% in Ghana or Tanzania, it was not surprising that about 7% of women felt intimidated to talk to providers. Meanwhile, 17%-36% of women across the three countries were sometimes concerned that the providers were not providing enough care for them but could not express their feelings. The UK quality care commission[19] defines respect and dignity in care as care "that treats care recipients as equals" who merely require support from care providers to be autonomous and independent[20]. This definition conforms with the 4th, 5th and 6th WHO standards for facility quality of care for mothers and newborns[14].

Abuse is a seen as a way of controlling clients to undermine their autonomy[11]. The assessment findings confirm that, across the three countries, physical, sexual, verbal and psychological abuses (where

services are withheld from women because of inability to pay informal/illegal charges) were experienced by women. These findings concur with reports of care providers “scolding” or “shouting at” women and verbalising words that were “unkind, brusque, rude, unsympathetic and uncaring” to them[21 22]. We believe that the 4.6%-6.6% of verbal abuses may represent gross under-reporting and that the challenge might be bigger. Also, providing RMC requires development of infrastructure. For instance, none of the three countries prohibits the use of birth companions but the set-up of the units did not allow for privacy or birth companions in these units.

Respectful care is also not merely the absence of abuses. Policies on the rights of maternity clients and for addressing abuse are not always present in facilities particularly in Tanzania. Even when they existed, providers were oblivious, or the enforcement was weak. A conducive policy atmosphere facilitates reporting of abuse cases. Our finding that 25% (Tanzania) to 60% (Bangladesh) of providers have not been trained on provision of dignified and RMC is unfortunate. For instance, Ghana reported the most advanced policy environment to identify and address abuses including a “whistle-blower” policy. Consequently, women in Ghana were the most likely to report of abuses. This suggests that addressing abuses in maternity care requires deliberate efforts to create conducive environments that address systems rather than individual failures that occasion “blame” – literally “A systems approach in a person-centred system”[23 24].

The WHO statement affirms every woman’s right to dignified, respectful healthcare, identifying an increasing need for more research to measure the burden and pervasiveness of disrespect and abuse in health facilities[1 21 22 25]. Vogel et al[25] added that poor measurement of respectful care may result from the lack of globally applicable terminologies.

Initiatives to improve respect around childbirth care are not new in these countries albeit on smaller scales: Bangladesh has implemented a Women Friendly Health Services Initiative in some facilities but Kurigram district did not benefit. Similarly, the Ifakara Research Institute in Tanzania is also implementing RMC interventions in some facilities. These have not permeated the healthcare systems. That quality affects outcomes is incontrovertible[26]. We believe that efforts to provide RMC must be intentional, goal-directed, measurable, backed by a strong political will, and should have robust systems of measurement and accountability[27].

This study has many strengths: first, using harmonised methods to assess the 43 facilities makes it one of the largest multi-country facility assessments from which data is amenable to direct cross-country comparisons. Secondly, the use of a variety of approaches including observations and data review increased the objectivity and improved understanding of how prevailing practices measure up to the standards at individual, structural and policy levels according to Freedman et al’s framework[28].

We had limitations: the cross-sectional design does not allow for attribution of causality and the short duration of contact with facilities limited the aspects of respectful care that was assessed. However, it was the most pragmatic design for many settings if it should not unduly interrupt caregiving. Women’s reported experiences and caregiver’s practices were subject to biases. Observing provider-client interactions may likely to make them go beyond what they usually do (Hawthorne effect). Consequently, inadequacies identified during the observations may represent a small dip in an abyss of inadequate respect in care. These findings may not be generalisable to all facilities in the respective countries because, due to UNICEF’s equity focus, assessed facilities were located in most under-served and least-developed districts which may imply selection biases. The assessment did not measure the

For peer review only

psychometric properties of any of the indicators and merely generated proposed candidate indicators for future testing.

This study contributes to amplifying women's voices, which is key to the values of the WHO/UNICEF-coordinated Quality, Equity, Dignity network and the recent petition on "What women want?"[29]. However, women's aversion to care that is not dignified or respectful, nuanced in their decision not to patronise these facilities in the future should alert health systems that community trust in facilities for delivery care should not be taken for granted. The Lancet Quality Commission advocates for measurement of client satisfaction on a systematic basis[9].

In conclusion, RMC provided and consequently experienced by women across the three countries had many good aspects that could form a foundation for quality improvement interventions. Health providers' accounts over-rated the respect and dignity in the care they provide to women way beyond what was directly observed. Critical gaps still exist which a systematic approach to implementation of RMC, as envisaged in the EMEN-QI initiative, should address. It calls for more rigorous measurement of efficacy of these measures as well as in-depth anthropological investigation to understand the drivers of respectful and dignified care to help develop corrective strategies and accountability mechanisms around these in a participatory manner. As a first step, respectful care must form a critical component of pre-service training in all countries. Community engagement through organizing for a systematic demand creation around respectful care, follow-up and inclusion in a holistic system response will be key. Periodic client-exit interviews, use of mobile Short Messaging Services, comment boxes or easy-touch buttons for client feedback will be useful but need to be coupled with capacity to retrieve these data, systematically analyse them and use them to inform changes. Health systems and facilities should be bold to empower an approachable community ombudsman to listen to client complaints and provide systematic feedback into the delivery RMC in facilities[30] . Until then, our mothers will not return to our facilities after their experiences with providers in facilities.

Author contributions

The study was designed by AM, NZ, EM, JW, and SA with technical input from CX. AM, NZ, EM, JW, SA, formed a team that managed and supported the respective countries to implement the evaluation. AM and NZ drafted the manuscript and obtained inputs from the co-authors: JW, EM, SA, CX. The revised manuscripts were proof-read by all authors who helped in finalising it. All co-authors (NZ, AM, EM, JW, SA) read the final manuscript and agreed to its submission for publication in this journal.

Acknowledgements

This work was conducted under the UNICEF-BMGF MNCH partnership. We also appreciate the contributions from Dr Priscilla Wobil (UNICEF Ghana), Masum Billah (ICDDR,B Bangladesh), Stella Kilima (NIMR, Tanzania), Francis Yeji (NHRC/GHS, Ghana), management and staff of our collaborating institutions at the International Centre for Diarrhoeal Disease Research, Bangladesh, Navrongo Research Centre of the Ghana Health Service, National Institute of Medical Research, Tanzania and Dr Tedbabe Hailegebriel, Dr Will Zeck and Seun Oyedele of UNICEF/HQ and the UNICEF country offices in the three countries. The multiple consultations with a wide range of stakeholders including

global/international experts and professionals in maternal and newborn health, ministries of health and partners in Bangladesh, Ghana and Tanzania. We also thank the mothers who participated in the design and provided data for this analysis.

Competing interests

All the authors declare no conflict of or competing interests.

Funding

The study was funded by the Bill and Melinda Gates Foundation through UNICEF headquarters, New York (Grant Number OPP1112117). The findings and conclusions in this report are those of the authors and do not represent the official position of these organizations.

Data Sharing Statement

The data analysed for this manuscript is part of the bigger EMEN assessment being undertaken by UNICEF. Access to the data will follow site-specific and national guidelines of the three countries and agreements with UNICEF HQ.

For peer review only

References

1. World Health Organization. The prevention and elimination of disrespect and abuse during facility-based childbirth. In: Department of Reproductive Health and Research WHO, ed. Geneva, Switzerland: World Health Organization, 2015.
2. Ng M, Misra A, Diwan V, et al. An assessment of the impact of the JSY cash transfer program on maternal mortality reduction in Madhya Pradesh, India. *Glob Health Action* 2014;7:24939. doi: 10.3402/gha.v7.24939
3. Countdown to 2030 Collaboration. Countdown to 2030: tracking progress towards universal coverage for reproductive, maternal, newborn, and child health. *Lancet* 2018;391(10129):1538-48. doi: 10.1016/S0140-6736(18)30104-1 [published Online First: 2018/02/06]
4. Kruk ME, Gage AD, Joseph NT, et al. Mortality due to low-quality health systems in the universal health coverage era: a systematic analysis of amenable deaths in 137 countries. *Lancet* 2018;392(10160):2203-12. doi: 10.1016/S0140-6736(18)31668-4 [published Online First: 2018/09/10]
5. Lawn JE, Blencowe H, Waiswa P, et al. Stillbirths: rates, risk factors, and acceleration towards 2030. *Lancet* 2016;387(10018):587-603. doi: 10.1016/S0140-6736(15)00837-5 [published Online First: 2016/01/23]
6. Ronsmans C, Graham WJ, Lancet Maternal Survival Series steering g. Maternal mortality: who, when, where, and why. *Lancet* 2006;368(9542):1189-200. doi: 10.1016/S0140-6736(06)69380-X
7. Doyle C, Lennox L, Bell D. A systematic review of evidence on the links between patient experience and clinical safety and effectiveness. *BMJ Open* 2013;3(1) doi: 10.1136/bmjopen-2012-001570 [published Online First: 2013/01/08]
8. Souza JP, Gulmezoglu AM, Vogel J, et al. Moving beyond essential interventions for reduction of maternal mortality (the WHO Multicountry Survey on Maternal and Newborn Health): a cross-sectional study. *Lancet* 2013;381(9879):1747-55. doi: 10.1016/S0140-6736(13)60686-8
9. Kruk ME, Gage AD, Arsenault C, et al. High-quality health systems in the Sustainable Development Goals era: time for a revolution. *Lancet Glob Health* 2018;6(11):e1196-e252. doi: 10.1016/S2214-109X(18)30386-3 [published Online First: 2018/09/10]
10. Larson E, Leslie HH, Kruk ME. The determinants and outcomes of good provider communication: a cross-sectional study in seven African countries. *BMJ Open* 2017;7(6):e014888. doi: 10.1136/bmjopen-2016-014888 [published Online First: 2017/07/05]
11. d'Oliveira AF, Diniz SG, Schraiber LB. Violence against women in health-care institutions: an emerging problem. *Lancet* 2002;359(9318):1681-5. doi: 10.1016/S0140-6736(02)08592-6 [published Online First: 2002/05/22]
12. Silal SP, Penn-Kekana L, Harris B, et al. Exploring inequalities in access to and use of maternal health services in South Africa. *BMC Health Serv Res* 2012;12:120. doi: 10.1186/1472-6963-12-120 [published Online First: 2012/05/23]
13. Small R, Yelland J, Lumley J, et al. Immigrant women's views about care during labor and birth: an Australian study of Vietnamese, Turkish, and Filipino women. *Birth* 2002;29(4):266-77. [published Online First: 2002/11/15]
14. World Health Organization. Standards for improving quality of maternal and newborn care in health facilities. Geneva, Switzwerland: World Health Organization, 2016.
15. de Bernis L, Kinney MV, Stones W, et al. Stillbirths: ending preventable deaths by 2030. *Lancet* 2016;387(10019):703-16. doi: 10.1016/S0140-6736(15)00954-X [published Online First: 2016/01/23]
16. Vedam S, Stoll K, Rubashkin N, et al. The Mothers on Respect (MOR) index: measuring quality, safety, and human rights in childbirth. *SSM Popul Health* 2017;3:201-10. doi: 10.1016/j.ssmph.2017.01.005 [published Online First: 2018/01/20]

For peer review only

17. Manu A, Arifeen S, Williams J, et al. Assessment of facility readiness for implementing the WHO/UNICEF standards for improving quality of maternal and newborn care in health facilities - experiences from UNICEF's implementation in three countries of South Asia and sub-Saharan Africa. *BMC Health Serv Res* 2018;18(1):531. doi: 10.1186/s12913-018-3334-0 [published Online First: 2018/07/11]
18. Bohren MA, Hunter EC, Munthe-Kaas HM, et al. Facilitators and barriers to facility-based delivery in low- and middle-income countries: a qualitative evidence synthesis. *Reprod Health* 2014;11(1):71. doi: 10.1186/1742-4755-11-71 [published Online First: 2014/09/23]
19. Care Quality Commission UK. Health and Social Care Act 2008 (Regulated Activities) Regulations 2014: Regulation 10. http://www.cqc.org.uk/guidance-providers/regulations-enforcement/regulation-10-dignity-respect.
20. World Health Organization; Organisation for Economic Co-operation and Development; and The World Bank. Delivering quality health services: a global imperative for universal health coverage. Geneva, Switzerland: World Health Organization Organisation for Economic Co-operation and Development and The World Bank., 2018.
21. Bohren MA, Vogel JP, Tuncalp O, et al. "By slapping their laps, the patient will know that you truly care for her": A qualitative study on social norms and acceptability of the mistreatment of women during childbirth in Abuja, Nigeria. *SSM Popul Health* 2016;2:640-55. doi: 10.1016/j.ssmph.2016.07.003 [published Online First: 2017/03/28]
22. Bohren MA, Vogel JP, Hunter EC, et al. The Mistreatment of Women during Childbirth in Health Facilities Globally: A Mixed-Methods Systematic Review. *PLoS Med* 2015;12(6):e1001847; discussion e47. doi: 10.1371/journal.pmed.1001847
23. National Academies of Sciences Engineering and Medicine. Crossing

the global quality chasm: Improving health care worldwide. Washington, DC, USA: National Academies of Sciences, Engineering, and Medicine, 2018.
24. Buckley R. World Development Report 2004: Making services work for poor people-Overview. Washington, DC, USA: The World Bank, 2003. http://documents.worldbank.org/curated/en/527371468166770790/, 2004.
25. Vogel JP, Bohren MA, Tuncalp, et al. Promoting respect and preventing mistreatment during childbirth. *BJOG* 2016;123(5):671-4. doi: 10.1111/1471-0528.13750 [published Online First: 2015/12/03]
26. Miller S, Lalonde A. The global epidemic of abuse and disrespect during childbirth: History, evidence, interventions, and FIGO's mother-baby friendly birthing facilities initiative. *Int J Gynaecol Obstet* 2015;131 Suppl 1:S49-52. doi: 10.1016/j.ijgo.2015.02.005
27. Van Belle S, Mayhew SH. Public accountability needs to be enforced -a case study of the governance arrangements and accountability practices in a rural health district in Ghana. *BMC Health Serv Res* 2016;16(1):568. doi: 10.1186/s12913-016-1836-1 [published Online First: 2016/10/13]
28. Freedman LP, Ramsey K, Abuya T, et al. Defining disrespect and abuse of women in childbirth: a research, policy and rights agenda. *Bull World Health Organ* 2014;92(12):915-7. doi: 10.2471/BLT.14.137869
29. White Ribbon Alliance. What Women Want: Demands for quality healthcare for women and girls. https://www.whiteribbonalliance.org/whatwomenwant/, Accessed November 2018.
30. World Health Organization and the United Nations Children’s Fund (UNICEF). Declaration of Astana: From Alma-Ata towards universal health coverage and the Sustainable Development Goals. Astana, Kazakhstan: World Health Organization, 2018.

For peer review only

List of tables

Table 1. Facility managers' report on the availability of guidelines for identifying, responding and preventing abuses and community involvement in ensuring quality of care

Guideline or policy parameter in health facilities	Number of responses (%)		
	Bangladesh N=15	Ghana N=16	Tanzania N=12
A process for identifying abuses of client by provider	11 (73.3%)	13 (81.3%)	6 (50.0%)
"Whistle-blower" policy for peer reporting under cover	Not recoded	15 (93.8%)	3 (25.0%)
Women are encouraged to report provider abuses	9 (60.0%)	16 (100.0%)	4 (33.3%)
Response to most recent abuse case (number of cases)	1 case	5 cases	1 case
- *Investigated and penal actions taken internally*	0 (0.0%)	4 (80.0%)	1 (100.0%)
- *Investigated within facility but referred for action*	0 (0.0%)	1 (20.0%)	0 (0.0%)
- *Referred to external bodies for investigation & sanction*	1 (100.0%)	0 (0.0%)	0 (0.0%)
Measures taken to prevent future abuses¥			
- *Counselling and verbal warning*	1 (100.0%)*	5 (100.0%)	1 (100.0%)
Involves community stakeholders in quality improvement efforts	8 (53.3%)	12 (75.0%)	6 (50.0%)
Women's support groups exist in the catchment population	5 (33.3%)	10 (62.5%)	2 (16.7%)

**In Bangladesh, they raise awareness and supervise the staff who perpetrated the act.* ¥*The assessment checked for other penal measures such as demotion of staff, reassignment/transfer, embargo on salary and interdiction but there was not reported in any of the facilities.*

For peer review only

Table 2. Care providers' report on availability of guidelines for respectful maternal and newborn care

Subject area	Providers' account on modalities for ensuring respectful maternity and newborn care	Number of responses (%)		
		Bangladesh N=158	Ghana N=86	Tanzania N=81
**Policies on women's rights**	Policy and procedure for addressing patient concerns	54 (41.1%)	61 (71.0%)	54 (66.7%)
	Policies on the rights of patients	83 (52.5%)	70 (81.6%)	57 (70.4%)
**Staff training on Respectful care**	Staff trained on how to treat childbearing women with respect	64 (40.5%)	60 (70.1%)	61 (75.0%)
**Staff rating of RMC provided in their facility**	Staff who rated their facility above 3 on a scale of 1 to 5 (5 being best) for care with respect and dignity they provide to women.	151 (95.6%)	86 (100.0%)	77 (95.1%)
**Identification and response to Abuse**	Process exist for identifying and reporting abuses during maternity and newborn care	46 (29.1%)	58 (67.8%)	41 (50.0%)
	Have encountered abuse victims in their maternity and newborn care practice	42 (26.6%)	28 (32.2%)	16 (19.8%)
	Staff who knew of 2 or more measures being implemented in their facilities to prevent abuses	31 (19.6%)	54 (62.7%)	57 (70.4%)
	Staff who mentioned 2 or more measures being implemented in their facilities to treat or rehabilitate victims of abuses	16 (10.1%)	17 (19.5%)	56 (69.0%)
	Community stakeholders' involvement in addressing disrespect and abuse of women during childbirth	49 (31.0%)	49 (57.5%)	27 (33.3%)
**Payment for services**	Women are expected to pay for normal deliveries	32 (20.3%)	7 (8.1%)	42 (51.9%)
	Women are expected to buy supplies for normal deliveries	132 (83.5%)	73 (85.1%)	61 (75.0%)
	Women are expected to pay before treatment in case of obstetric emergencies	19 (12.0%)	9 (10.3%)	27 (33.3%)

For peer review only

Table 3. Women's report of abuses, provider attitudes and overall satisfaction with care provided

Aspect of care	Modality for which women's perspective around facility experiences was assessed	Number of responses (%)		
		Bangladesh N=295	Ghana N=381	Tanzania N=173
Abuses during care-giving	Physical abuse from providers at the facility	6 (2.0%)	9 (2.4%)	2 (1.2%)
	Verbal abuse from providers at the facility	19 (6.4%)	25 (6.6%)	8 (4.6%)
	Sexual abuse from providers at the facility	2 (0.6%)	2 (0.7%)	1 (0.6%)
Provider relationship with caregivers	Their concerns were considered by providers in caring for them	224 (75.9%)	352 (92.4%)	108 (62.4%)
	Providers were responsive when asked for support	262 (88.8%)	366 (96.1%)	161 (93.1%)
	They were treated with respect by providers	266 (90.2%)	367 (96.1%)	168 (97.1%)
Satisfaction with care	Satisfied with attitude of providers	271 (91.7%)	353 (92.7%)	161 (93.1%)
	Satisfied with information received from providers on breastfeeding	191 (64.8%)	338 (88.7%)	132 (76.3%)
	Satisfied with information received from providers on family planning	38 (12.9%)	208 (54.6%)	77 (44.5%)

Table 4. Assessor observations of the provider attitudes during maternity care for women

Aspect of care	Modalities of care that were observed by assessors	Number of responses (%)		
		Bangladesh N=387	Ghana N=134	Tanzania N=120
Client requests	Women who made request for anything	14 (3.7%)	4 (3.0%)	4 (3.3%)
	- Those whose requests were refused with respect	1 (6.7%)	4 (100.0%)	3 (75.0%)
	Women who requested for a birth companion	286 (73.9%)	0 (0.0%)	2 (1.7%)
Payments at facility and effect on care	Women/families paid monies for some services	164 (42.4%)	19 (14.2%)	61 (50.8%)
	Women/families who complained about the payment	45 (11.6%)	1 (0.8%)	22 (18.3%)
	Women for whom services were withheld due to inability to pay	2 (0.5%)	0 (0.0%)	13 (10.8%)

For peer review only

Legend of figures

Figure 1. Availability of client toilets with soap and water for handwashing and its cleanliness

Figure 2. Women's reported experience of care at maternity units

Figure 3a-3d. Assessors' observations on women's maternity care experiences during provider interactions in health facilities

Figure 4. Mothers' satisfaction with care received in the health facilities during childbirth in the 3 countries

For peer review only

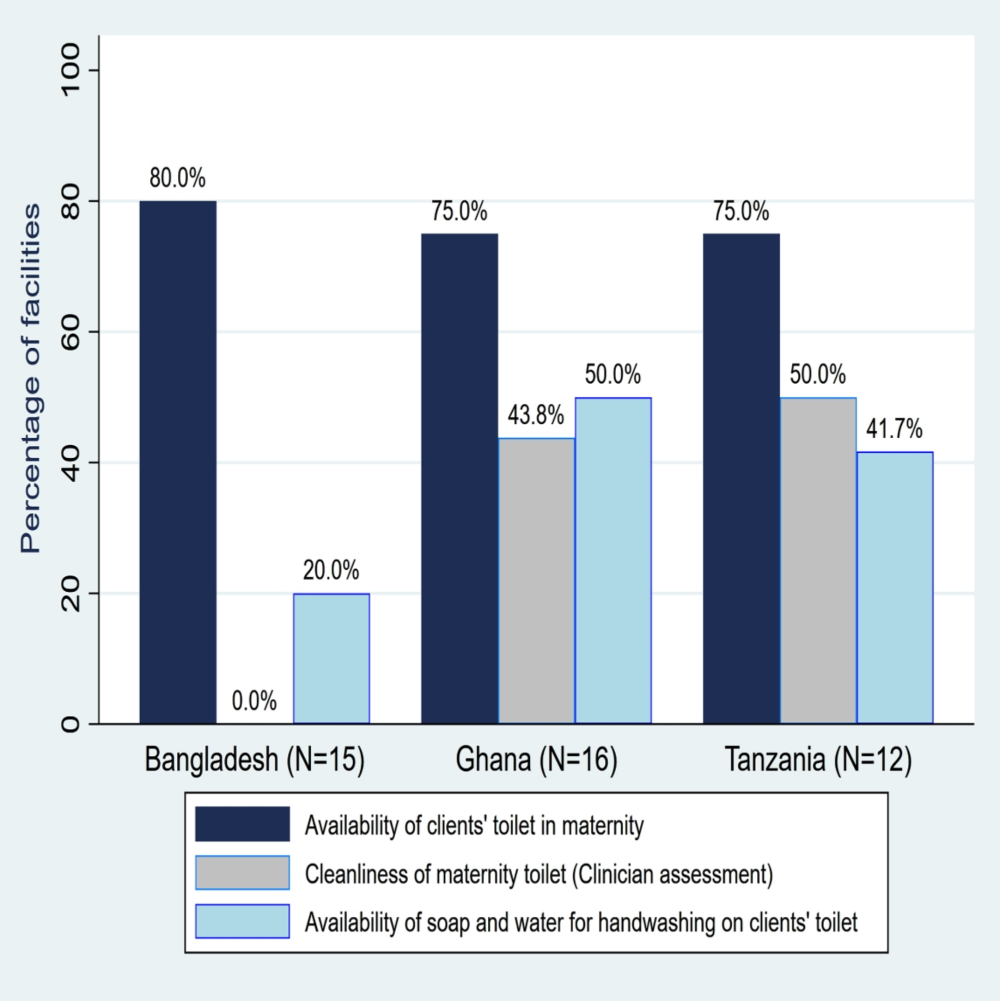

Figure 1. Availability of client toilets with soap and water for handwashing and its cleanliness

90x90mm (600 x 600 DPI)

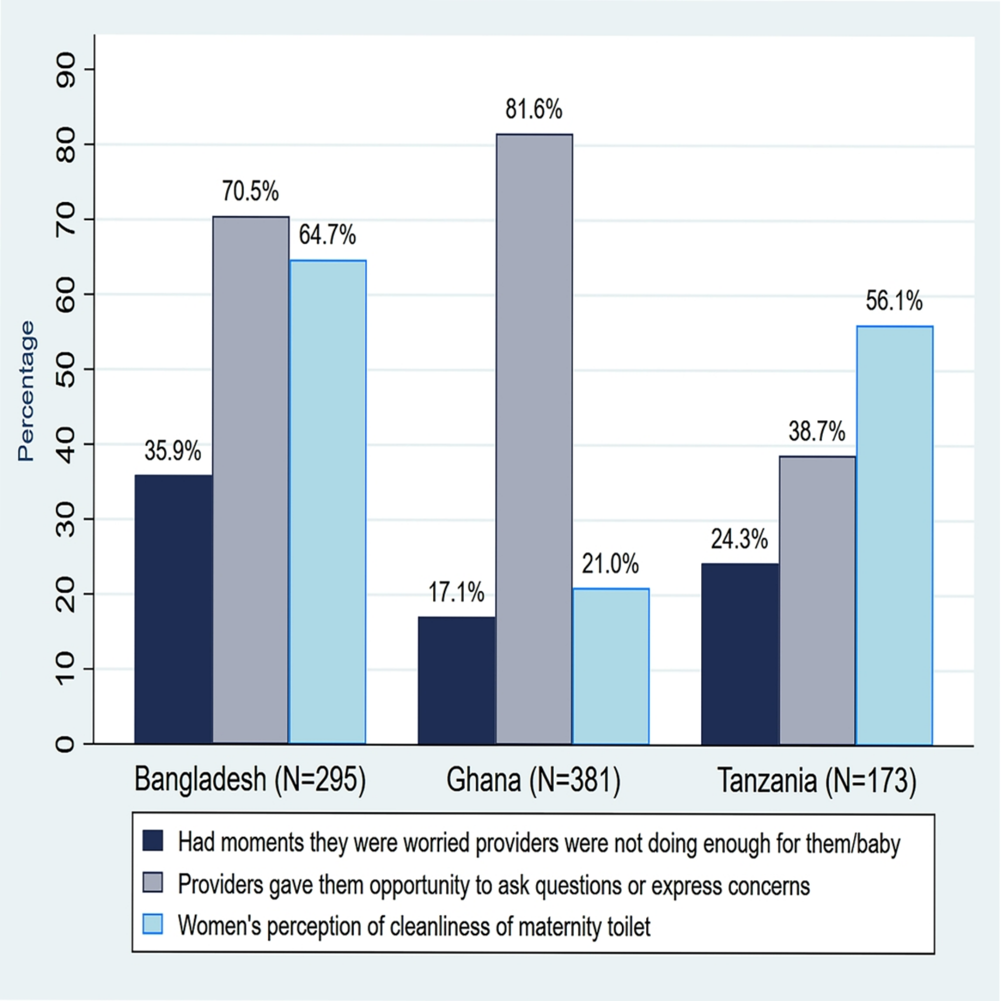

Figure 2. Women's reported experience of care at maternity units

90x90mm (600 x 600 DPI)

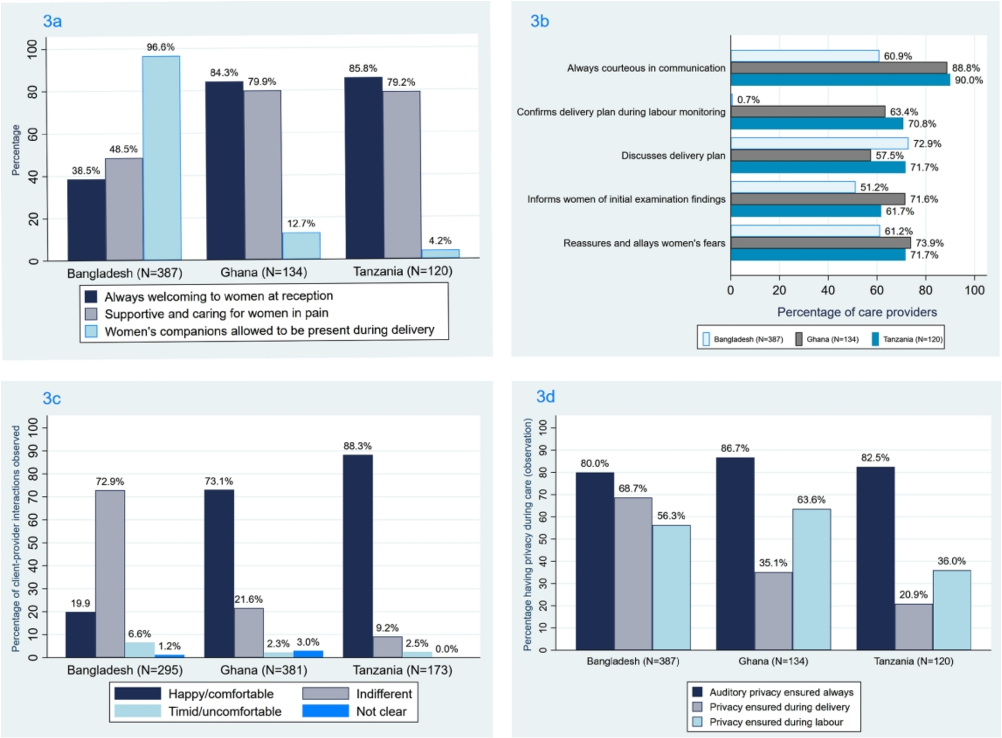

Figure 3a-3d. Assessors' observations on women's maternity care experiences during provider interactions in health facilities

90x66mm (600 x 600 DPI)

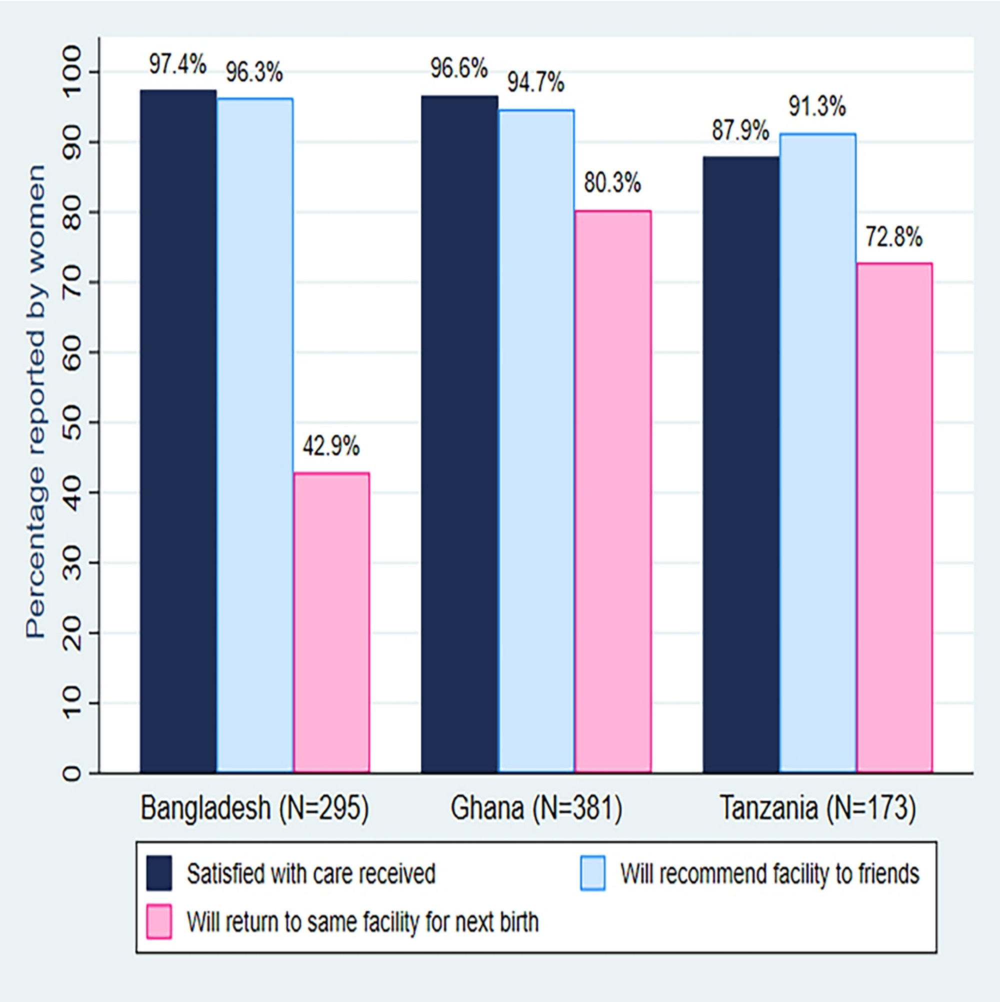

Figure 4. Mothers' satisfaction with care received in the health facilities during childbirth in the 3 countries

90x90mm (600 x 600 DPI)

STROBE guideline checklist

**STROBE guidelines checklist**	**Page number**
Title	1
Abstract	2
**Introduction**	**4**
Objectives	4
**Methods**	
Study design	4
Setting	5
Participants	5
Variables	6
Data sources / measurement	6
Bias	6
Study size	6
Quantitative variables	6
Statistical methods	7
**Results**	
Participants	8
Descriptive data	8
Outcome data	8-11
Main results	8-11
Other analysis	Not applicable
**Discussion**	
Key results	11
Limitations	12
Interpretation	11-12
Generalizability	12
**Other information**	
Funding	13

For peer review only
